# Identifying Key Genes Involved in Axillary Lymph Node Metastasis in Breast Cancer Using Advanced RNA-Seq Analysis: A Methodological Approach with GLMQL and MAS

**DOI:** 10.3390/ijms25137306

**Published:** 2024-07-03

**Authors:** Mostafa Rezapour, Robert Wesolowski, Metin Nafi Gurcan

**Affiliations:** 1 Center for Artificial Intelligence Research, Wake Forest University School of Medicine, Winston-Salem, NC 27101, USA; mgurcan@wakehealth.edu; 2 Division of Medical Oncology, James Cancer Hospital and the Ohio State University Comprehensive Cancer Center, Columbus, OH 43210, USA; robert.wesolowski@osumc.edu

**Keywords:** breast cancer, axillary lymph node metastasis (ALNM), RNA sequencing (RNA-Seq), gene expression analysis, generalized linear models, quasi-likelihood F test

## Abstract

Our study aims to address the methodological challenges frequently encountered in RNA-Seq data analysis within cancer studies. Specifically, it enhances the identification of key genes involved in axillary lymph node metastasis (ALNM) in breast cancer. We employ Generalized Linear Models with Quasi-Likelihood (GLMQLs) to manage the inherently discrete and overdispersed nature of RNA-Seq data, marking a significant improvement over conventional methods such as the *t*-test, which assumes a normal distribution and equal variances across samples. We utilize the Trimmed Mean of M-values (TMMs) method for normalization to address library-specific compositional differences effectively. Our study focuses on a distinct cohort of 104 untreated patients from the TCGA Breast Invasive Carcinoma (BRCA) dataset to maintain an untainted genetic profile, thereby providing more accurate insights into the genetic underpinnings of lymph node metastasis. This strategic selection paves the way for developing early intervention strategies and targeted therapies. Our analysis is exclusively dedicated to protein-coding genes, enriched by the Magnitude Altitude Scoring (MAS) system, which rigorously identifies key genes that could serve as predictors in developing an ALNM predictive model. Our novel approach has pinpointed several genes significantly linked to ALNM in breast cancer, offering vital insights into the molecular dynamics of cancer development and metastasis. These genes, including *ERBB2*, *CCNA1*, *FOXC2*, *LEFTY2*, *VTN*, *ACKR3*, and *PTGS2*, are involved in key processes like apoptosis, epithelial–mesenchymal transition, angiogenesis, response to hypoxia, and KRAS signaling pathways, which are crucial for tumor virulence and the spread of metastases. Moreover, the approach has also emphasized the importance of the small proline-rich protein family (SPRR), including *SPRR2B*, *SPRR2E*, and *SPRR2D*, recognized for their significant involvement in cancer-related pathways and their potential as therapeutic targets. Important transcripts such as *H3C10*, *H1-2*, *PADI4*, and others have been highlighted as critical in modulating the chromatin structure and gene expression, fundamental for the progression and spread of cancer.

## 1. Introduction

Female breast cancer continues to represent a significant portion of cancer cases, with an age-adjusted rate of new cases at 129.4 per 100,000 women per year based on 2017–2021 data and a death rate of 19.3 per 100,000 women per year from 2018–2022 [1,2]. The lifetime risk of developing breast cancer is approximately 13.0%, based on data from 2017–2019, reflecting its widespread prevalence [1,2]. As of 2021, there were approximately 3,972,256 women in the United States who had been diagnosed with breast cancer at some point in their lives. The overall 5-year relative survival rate for female breast cancer stands at 91.2%, though survival rates vary significantly by the stage at diagnosis; notably, localized cases identified at stage 1 exhibit a 5-year relative survival rate of 99.6% [1,2]. In 2024, it is estimated that there will be 310,720 new cases of female breast cancer, accounting for 15.5% of all new cancer cases, and 42,250 deaths, comprising 6.9% of all cancer deaths. These projections highlight the significant impact of breast cancer within the broader spectrum of cancer incidence and mortality [3].

Breast cancer remains a prominent public health concern as the second leading cause of cancer-related mortality among women in the U.S., surpassed only by lung cancer [4,5]. Central to the clinical management of breast cancer is the assessment of axillary lymph node metastasis (ALNM), a pivotal factor in the TNM (tumor, node, metastasis) staging system. This assessment crucially influences staging, treatment decisions, and prognosis by integrating the number and size of metastatic lymph nodes, thereby guiding therapeutic strategies [6].

The presence of ALNM is closely linked to poorer disease-free and overall survival rates, as well as increased chances of recurrence, making its accurate detection and evaluation a cornerstone of effective breast cancer management [7]. Traditionally, ALNM assessment has depended on invasive histopathological examinations. These methods not only delay treatment initiation [8,9,10] but also depend heavily on the availability of significant clinical expertise [11,12]. Additionally, they are associated with complications such as lymphedema, increased risk of infections, cording, and longer post-operative recovery, particularly in procedures like sentinel lymph node biopsy and axillary lymph node dissection. Given these limitations, there has been a growing shift towards more accurate, efficient, and less invasive techniques, facilitated by advancements in precision medicine and molecular oncology [13].

Despite advancements, challenges remain, particularly in detecting metastases that are not visible on traditional imaging techniques like ultrasound. These occult metastases are difficult to biopsy and pose significant hurdles in clinical decision-making [14,15,16]. Integrating multi-omics data, including mRNA, miRNA, and DNA methylation, has shown promising results in improving the accuracy of ALNM predictions, as evidenced by various studies utilizing artificial intelligence (AI) and machine learning (ML) models on large datasets like those from TCGA [17,18,19,20]. However, a recurrent issue in these studies is the statistical treatment of RNA-Seq data. Some research treats RNA-Seq data as continuous, which overlooks its intrinsic discrete and overdispersed nature. Other studies lack detailed statistical analysis, leaving critical data assumptions unaddressed. Even in cases where RNA-Seq data are recognized as discrete and overdispersed, the normalization methods used, such as fragments per kilobase of transcript per million mapped reads (FPKM) [21], do not adequately adjust for gene length or total read count, potentially skewing the gene expression measurements and affecting the study outcomes. Here are a few examples illustrating these points:

Kim et al. [22] investigated the metastatic progression of estrogen receptor-positive and HER2-negative breast cancer by comparing gene expression in normal breast tissue, primary tumors, and lymph node metastases. They utilized TopHat [23] and Cufflinks [24] for mapping and quantifying RNA-Seq data, estimating expression levels as fragments per kilobase of exon per million mapped reads (FPKM). Their results identified 2186 differentially expressed genes indicative of the transition from normal tissue to primary cancer and to metastasis, with notable changes in genes linked to cell adhesion and immune response. However, their dependence on FPKM for quantification, which fails to adjust for gene length bias, or the total read count, might compromise the accuracy of their gene expression measurements.

Liang et al. [25] conducted an extensive analysis of RNA sequencing data from treatment-naïve breast cancer patients to identify molecular markers predicting non-sentinel lymph node (NSLN) status in patients with metastatic sentinel lymph nodes (SLN). Their study involved extracting RNA from paraffin-embedded SLN samples, constructing libraries with the Illumina TruSeq RNA Sample Preparation Kit, and sequencing on a HiSeq 2000 Genome Analyzer. Gene expression was processed using TopHat [23] and Cufflinks [24] aligned to the human genome (hg19) and normalized using FPKM. The analysis included unsupervised hierarchical clustering to correlate gene expression with NSLN status and differential expression analysis using Cuffdiff [26] to identify uniquely expressed genes in NSLN negative or positive groups. However, their use of FPKM for normalization does not address potential biases such as gene length and the total number of mapped reads.

Dihge et al. [17] analyzed RNA sequencing (RNA-Seq) data combined with clinicopathological features to predict axillary lymph node metastasis in breast cancer, utilizing a cohort from the Sweden Cancerome Analysis Network–Breast (SCAN-B) which included RNA-Seq profiles for 3023 patients. They excluded low-quality reads and quantified gene expression using FPKM values, which were log2 transformed to enhance analytical reliability and normalize data across samples. To build predictive models, they applied generalized boosted regression models (GBMs) and other machine learning techniques, rigorously validated via cross-validation to ensure robustness. This methodology, especially the use of GBMs and the integration of RNA-Seq with clinical variables, markedly enhanced predictive accuracy for lymph node metastasis. However, it is important to note that FPKM, despite its common usage for RNA-Seq normalization, does not adjust for gene length and total read count, potentially limiting the accuracy of the expression measurements.

Note that studies by Kim et al. [22], Liang et al. [25], and Dihge et al. [17] utilized TopHat [23], and Cufflinks [24] for RNA-Seq analysis. While these tools are foundational, they do not address the discreteness and overdispersion as effectively as later tools like EdgeR [27], which employ models such as the negative binomial distribution to tackle these statistical challenges.

ALNM serves as a critical prognostic factor in breast cancer, significantly influencing both treatment decisions and patient outcomes [28]. Early detection of metastasis is crucial, as it often signals a higher risk of systemic disease spread, thereby affecting survival rates and shaping treatment protocols [29]. Traditional assessment methods frequently fail to capture subtle yet crucial gene expression variations due to limitations in the statistical models commonly used [30]. In contrast, our methodology employs generalized linear models with quasi-likelihood F-tests (GLMQL) in conjunction with magnitude altitude scoring (MAS) [31,32]. This approach not only assesses the statistical significance of gene expression changes but also evaluates their biological impact, providing a deeper insight into the molecular dynamics critical for ALNM. Such detailed analysis is essential in breast cancer, where minor variations in gene expression can significantly influence disease progression and management.

This advanced analytical strategy has been previously validated in our viral infection studies, where it effectively identified key biomarkers for early detection and targeted treatment strategies [31,32]. To enhance the reliability of our data, we utilize the trimmed mean of M-values (TMM) for normalization [33], which adjusts for compositional differences across RNA-Seq libraries, thereby ensuring more accurate gene expression measurements. By focusing on an untreated patient cohort, we obtain a pristine dataset, devoid of confounding treatment effects, that allows for a clearer understanding of the genetic underpinnings of ALNM. Additionally, our study specifically targets protein-coding genes, directly involved in cellular functions and disease mechanisms, ensuring that our findings are both biologically pertinent and therapeutically relevant. We further extend our analysis by conducting a comprehensive gene ontology (GO) and GSEA Hallmark set analysis. This not only categorizes significant genes into biological processes, cellular components, and molecular functions but also highlights the key pathways disrupted by ALNM, offering valuable insights into potential therapeutic targets and intervention strategies. The following key contributions highlight the distinctive aspects of our work:

1. **Implementation of Generalized Linear Models with Quasi-Likelihood F-tests and Magnitude Altitude Scoring (GLMQL-MAS) [31,32]:** Our application of GLMQL-MAS addresses the challenges posed by the discrete and overdispersed nature of RNA-Seq data. This approach enhances our capability to accurately identify and analyze key genes associated with ALNM, surpassing the limitations of traditional statistical methods.
2. **Utilization of Trimmed Mean of M-values (TMM) for Normalization [33]:** We adopt TMM normalization to correct for library-specific compositional differences, significantly improving the accuracy of gene expression measurements compared to conventional methods such as TPM [34] or FPKM [21] normalizations.
3. **Focus on Untreated Patient Cohort:** To the best of our knowledge, this is the first study that analyzes RNA-Seq data from a cohort of untreated breast cancer patients specifically for ALNM research. This unique focus allows us to observe the natural tumor environment without the confounding effects of prior treatments, enhancing the reliability of our findings in understanding the genetic underpinnings of lymph node metastasis.
4. **Exclusive Analysis of Protein-Coding Genes:** By concentrating on protein-coding genes, our research targets those genomic elements most directly involved in cellular functions and disease mechanisms, ensuring that our findings are biologically significant and directly applicable to potential therapeutic interventions. Although this approach leaves out non-coding regions, which may include fragments involved in gene translation (such as microRNAs) and potentially influence gene expression, focusing on protein-coding genes provides clearer insights into disease mechanisms and therapeutic targets.
5. **Comprehensive Gene Ontology (GO)** [35] **and GSEA Hallmark Set** [36,37] **Analysis:** Our study extends beyond traditional gene expression analysis by employing comprehensive GO and GSEA Hallmark set analyses. This approach allows us to categorize significant genes and identify disrupted biological pathways, providing actionable insights that could lead to the development of markers for early detection and targeted treatment strategies.

These methodological and analytical advancements significantly enhance the accuracy and clinical relevance of RNA-Seq data analysis for breast cancer diagnostics and treatment.

## 2. Results

### 2.1. Applying GLMQL-MAS for the Analysis of Axillary Lymph Node Metastasis

#### Differential Expression Analysis

**Case 1: 65 ALNM− Versus 42 ALNM+:**

**Figure 1:**
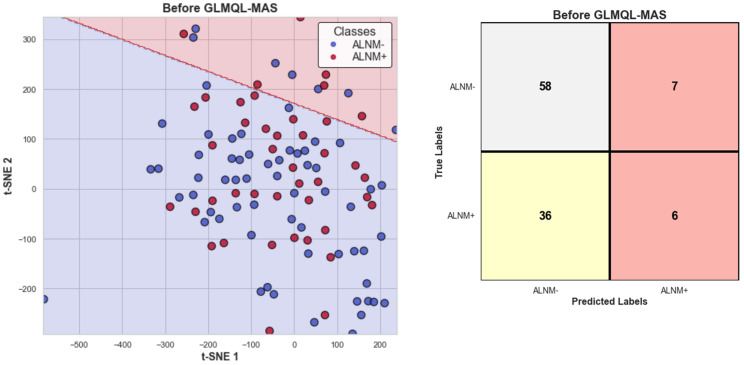
T-SNE projection of TMM-normalized gene expression data, illustrating the logistic regression decision boundary between ALNM− and ALNM+ samples. The red background represents areas predicted as ALNM+, while the blue background indicates ALNM−. The accompanying confusion matrix showcases the challenge of distinguishing samples without the application of GLMQL-MAS filtering.

Figure 1 illustrates the projection of all subjects’ TMM-normalized gene expression onto a two-dimensional plane, defined by t-SNE [38] coordinates Tsne1 and Tsne2. To assess the separability of ALNM− and ALNM+ samples within this projection, a logistic regression model was applied to the entire dataset. It is important to note that the purpose of this logistic regression analysis is not prediction; instead, it serves to demonstrate that, without appropriate filtering through the GLMQL-MAS process, the gene expression data become diluted, rendering the samples indistinguishable. Figure 1 presents both the logistic regression decision boundary and the corresponding confusion matrix, highlighting this effect.

**Figure 2:**
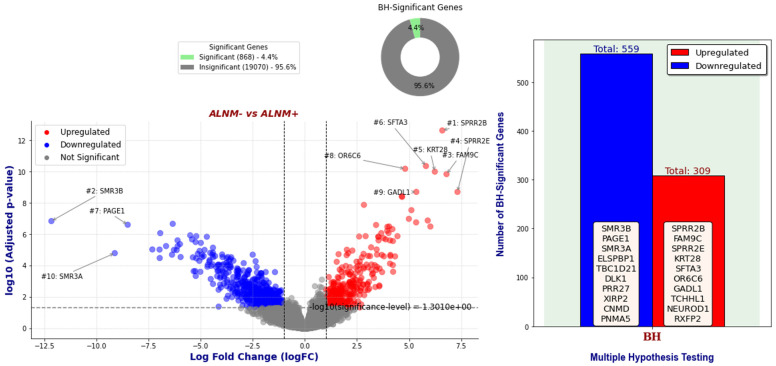
Volcano plots showcasing BH-significant genes (with threshold of |LogFC|>1) identified by the GLMQL-MAS system in the comparison of 42 ALNM+ samples against 65 ALNM− samples, highlighting 309 upregulated and 559 downregulated genes.

Figure 2 presents a volcano plot of genes identified as BH-significant through the GLMQL-MAS system, comparing 42 ALNM+ samples against 65 ALNM− samples. A threshold of |LogFC|>1 was applied to focus on genes with substantial expression changes. Notably, out of all analyzed genes, 868 (4.4%) met the criteria for BH significance. Among these, 309 genes were upregulated and 559 were downregulated, as determined by the GLMQL-MAS analysis. Figure S1 illustrates the hierarchical clustering heatmap of all samples using the top 100 GLMQL-MAS selected genes, which were filtered with a threshold of LogFC>1.

**Figure 3:**
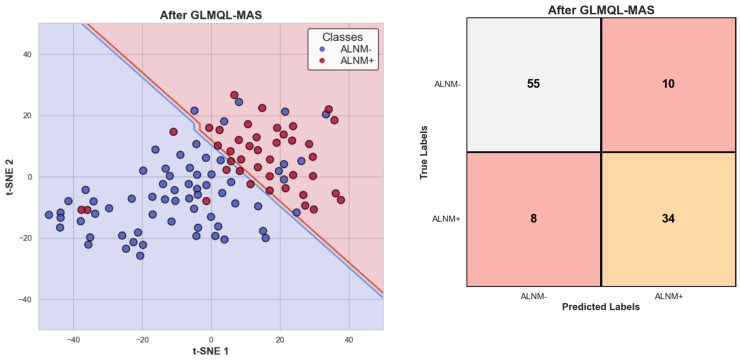
T-SNE projection using only GLMQL-MAS BH-significant genes, with the logistic regression decision boundary indicating improved separability between ALNM+ and ALNM− samples. The red background represents areas predicted as ALNM+, while the blue background indicates ALNM−. The included confusion matrix further illustrates the efficacy of the GLMQL-MAS filtering process.

Figure 3 displays the projection of all subjects on TSNE1 and TSNE2 axes, this time considering only GLMQL-MAS BH-significant genes. A logistic regression model was again applied, using TSNE1 and TSNE2 as predictors, to evaluate the separability of the denoised data. The corresponding confusion matrix and the logistic regression decision boundary are shown in Figure 3. Together, Figure 1 and Figure 3 demonstrate the efficacy of the GLMQL-MAS system in enhancing data separability within a non-predictive framework, where logistic regression is utilized not for prediction but to illustrate separability.

**Figure 4:**
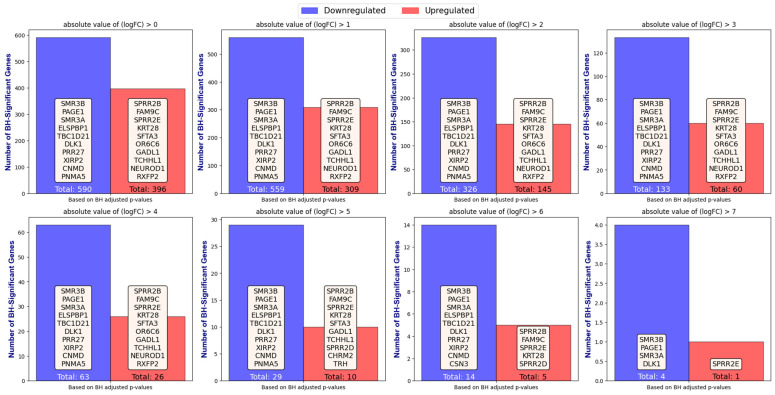
The figure demonstrates the robustness and independence of the GLMQL-MAS methodology from the |LogFC| threshold, emphasizing its capability to consistently select genes that maintain the largest possible values for both |LogFC| and the absolute log(BH-adjusted *p*-value), ensuring significant and impactful gene identification.

Figure 4 illustrates the number of upregulated and downregulated BH-significant genes identified by the GLMQL-MAS, showcasing the top 10 genes for each category as the upper threshold for the absolute value of log fold change (|LogFC|) varies between 0 and 7. This variation signifies that when |LogFC|>n, we define upregulated genes as having LogFC>n and downregulated genes as having LogFC<−n. The purpose of this figure is to demonstrate the stability and independence of the GLMQL-MAS from the LogFC threshold. It consistently selects the top genes to achieve the optimal balance, aiming for the largest possible values for both |LogFC| and the absolute value of the log(BH-adjusted *p*-value), thereby maximizing the significance and effect size of the identified genes simultaneously. Figures S2 and S3 further demonstrate this stability, showing consistent results when the GLMQL-RMAS is applied to raw *p*-values, and even when a Bonferroni correction is used instead of the Benjamini–Hochberg method in the GLMQL-MAS.

**Figure 5:**
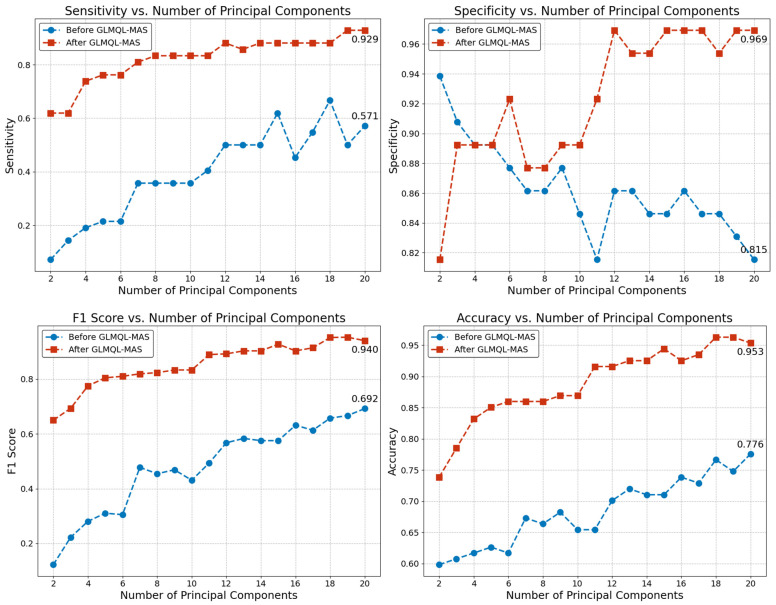
This figure evaluates the performance of a logistic regression model classifying samples as ALNM+ or ALNM− based on principal components derived from gene expression data. It compares metrics before and after applying the GLMQL-MAS methodology to highlight its effectiveness in refining gene selection. The analysis shows improvements in sensitivity, specificity, F1 score, and accuracy across principal components ranging from 2 to 20. The goal is not to develop a predictive model but to demonstrate the enhanced separation of disease states and the utility of GLMQL-MAS in biological data interpretation.

Figure 5 illustrates the effectiveness of the GLMQL-MAS methodology in enhancing the discrimination capabilities of a logistic regression model used for analyzing gene expression data. This approach does not aim to develop a predictive model but rather to demonstrate the distinction between ALNM+ and ALNM− samples using principal components derived from the entire gene dataset (before GLMQL-MAS) and from the gene subset refined by GLMQL-MAS (after GLMQL-MAS).

The analysis clearly shows significant improvements in model performance metrics such as sensitivity, specificity, F1 score, and accuracy across a range of principal components from 2 to 20. These enhancements highlight the capability of the GLMQL-MAS selected genes to more effectively separate the disease states, suggesting that this approach refines the gene set in a way that better captures the underlying biological differences between ALNM+ and ALNM− groups.

For sensitivity (top left panel), using more principal components results in a marked improvement in the model’s ability to correctly identify ALNM+ samples. Sensitivity values rise from a baseline of approximately 0.571 to a peak of 0.929. This increase suggests that the selected genes after GLMQL-MAS processing provide a more refined and effective set for distinguishing between disease states. The F1 score (bottom left panel) also shows significant enhancement post GLMQL-MAS, starting from 0.692 and reaching up to 0.940, reflecting a balanced measure of precision and recall provided by the gene subset refined through GLMQL-MAS.

To further explore the discriminative power of the genes identified by our GLMQL-MAS system, we employed linear discriminant analysis (LDA) [39] as a dimensionality reduction technique. Given that our analysis involves only two classes, ALNM+ and ALNM−, the use of LDA allows us to project the high-dimensional gene expression data onto a single dimension (LDA1) for a more straightforward visualization of class separability. The rationale behind selecting LDA1 lies in its ability to maximize the separation between the two groups by considering the variance between classes while minimizing the variance within each class. This approach is particularly useful in highlighting the distinct molecular signatures of ALNM+ versus ALNM− samples, which can be obscured in higher-dimensional spaces.

Figure S4 presents the LDA1 projection, showcasing the separation of ALNM+ from ALNM− samples using the top-n GLMQL-MAS selected genes, where n varies from 20 to 100. Remarkably, at n = 100, we achieve a perfect (100%) separation between the ALNM+ and ALNM− samples. This demonstrates the robust discriminative capability of the top-100 GLMQL-MAS selected genes, underscoring their potential relevance in understanding the molecular basis of lymph node metastasis in breast cancer.

Figure S5 illustrates the logistic regression decision boundaries using only TSNE1 and TSNE2 coordinates of samples projected with the top-n GLMQL-MAS selected genes for n = 100, n = 200, and n = 300. This figure highlights the efficacy of the GLMQL-MAS gene selection process in differentiating ALNM+ from ALNM− samples.

**Case 2. Random Sampling of 42 ALNM− to Compare Against 42 ALNM+:**

Following the random selection of 42 lymph node negative cases and contrasting them with 42 positive cases and repeating this process 500 times as described in Section 4.

**Figure 6:**
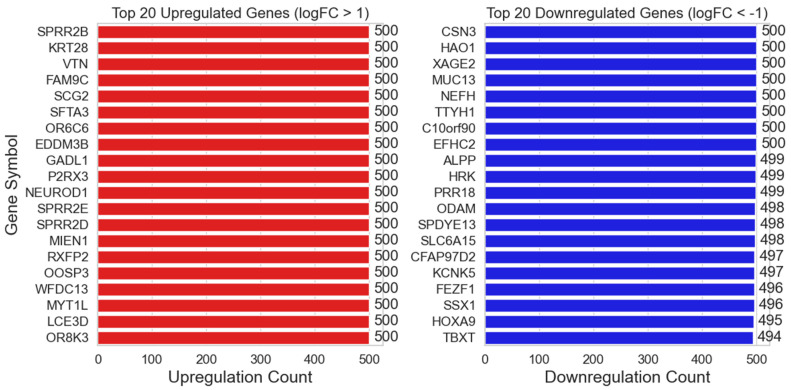
Distribution of the Top 20 Genes by BH-Significance Occurrence Using GLMQL-MAS. This figure illustrates the top-20 genes that achieved the highest frequency of BH-significance in an analysis of 500 iterations, with |LogFC| upper thresholds set at 1. It highlights the genes that consistently demonstrate significant differential expression in lymph node metastasis of breast cancer.

Figure 6 displays the top 20 genes with the highest number of BH-significance occurrences via GLMQL-MAS when the |LogFC| upper threshold is set at 1. Figure S6 displays the top 10 genes with the highest number of BH-significance occurrences via GLMQL-MAS when the |LogFC| upper threshold is set at 1, 2, 3, and 4.

### 2.2. Comprehensive Gene Ontology (GO) and Gene Set Enrichment Analysis (GSEA) of Hallmark Gene Sets in Lymph Node Metastasis of Breast Cancer

**Case 1. 65 ALNM− Versus 42 ALNM+:**

**Figure 7:**
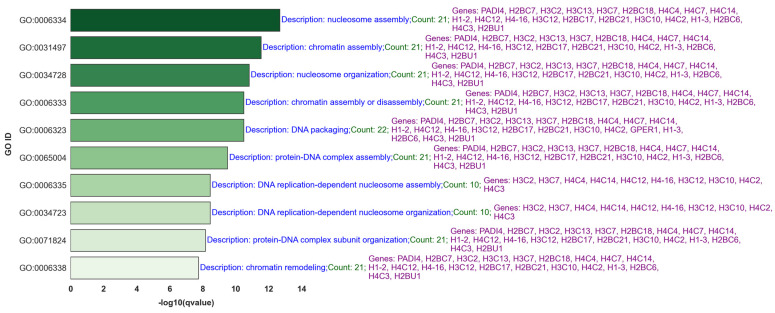
Top 10 GO Processes Related to Lymph Node Metastasis. This figure details the GO processes most intimately connected with the pathology of lymph node metastasis, providing insights into the molecular functions and cellular components affected.

**Figure 8:**
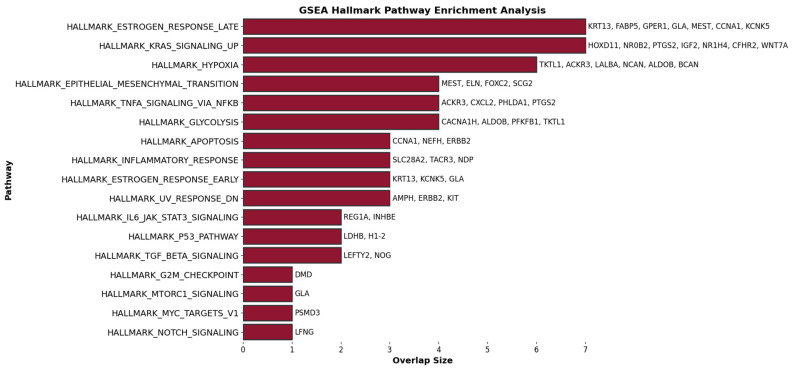
This figure illustrates a selection of the significant pathways identified from the GSEA using Hallmark gene sets, highlighting the predominant biological mechanisms influenced by the GLMQL-MAS selected genes between 42 ALNM positive (ALNM+) and 65 ALNM negative (ALNM−) breast cancer samples.

Figure 7 showcases the top 10 q-value-based significant gene ontology (GO) processes most closely associated with lymph node metastasis, focusing on upregulated BH-significant genes selected through the GLMQL-MAS methodology (Figure S7 shows the top 40). Figure S8 presents the results of the gene set enrichment analysis (GSEA) using Hallmark gene sets, further illustrating the key biological pathways influenced by these genetic changes in the context of lymph node metastasis. Figure 8 visualizes a selection of these pathways.

**Case 2. Random Sampling of 42 ALNM− to Compare Against 42 ALNM+:**

**Figure 9:**
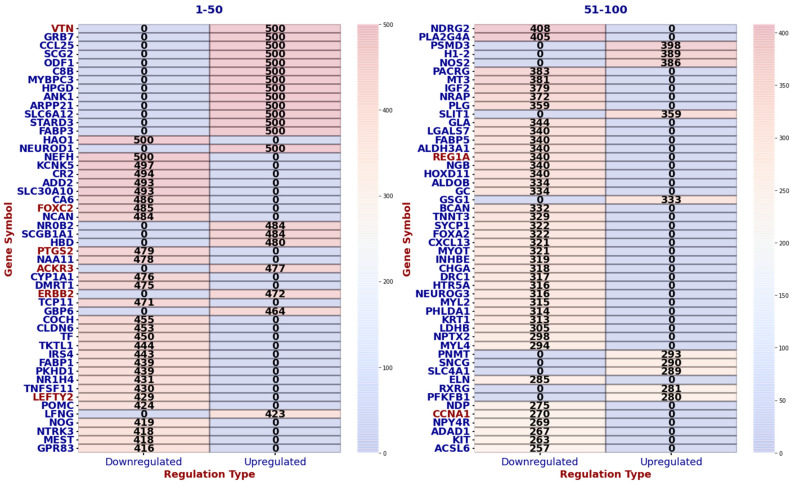
Top 100 consistent genes in random selections meeting GLMQL-MAS criteria displayed in one of the 50 GSEA Hallmark sets, specifically highlighting the key genes in cancer progression and metastasis in red.

Figure S9 presents the top q-value-based significant gene ontology (GO) processes for BH-significant upregulated genes, which were identified as being BH-significant and upregulated in at least 250 instances. Figure S10 showcases the results of the gene set enrichment analysis (GSEA) using Hallmark gene sets for all GLMQL-MAS selected genes, further detailing the key biological pathways influenced by these genetic variations in the context of lymph node metastasis. Figure 9 displays the top 100 genes in terms of consistency in upregulation or downregulation in random selections that meet the GLMQL-MAS criteria, as they appear in one of the 50 GSEA Hallmark sets.

#### Common Genes and GO and GSEA Hallmark Processes in Cases 1 and 2

**Figure 10:**
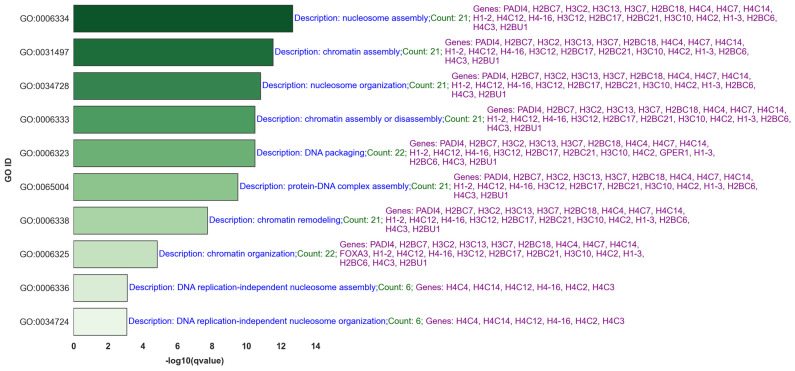
This figure displays common gene ontology (GO) processes and their associated upregulated BH-significant genes from analyses of 42 ALNM+ samples compared to ALNM− samples in two scenarios: Case 1, comparing 65 ALNM− versus 42 ALNM+; and Case 2, involving a random sampling of 42 ALNM− to compare against the same 42 ALNM+.

**Figure 11:**
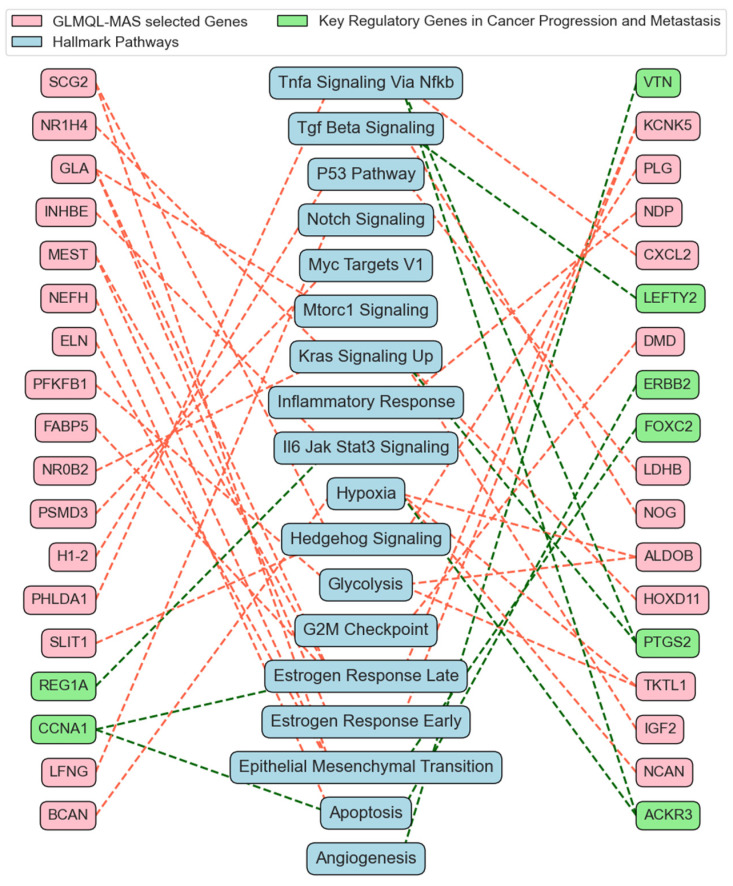
This figure illustrates a network that maps the most relevant Hallmark pathways to breast cancer, highlighting the common GLMQL-MAS selected genes from both Case 1 and Case 2.

Figure 10 displays the common gene ontology (GO) processes and their associated upregulated BH-significant genes from comparative analyses between 42 ALNM+ samples and ALNM− samples across two cases: Case 1, 65 ALNM− versus 42 ALNM+, and Case 2, random sampling of 42 ALNM− to compare against 42 ALNM+. Figure S11 showcases the results of the gene set enrichment analysis (GSEA) using Hallmark gene sets for the common genes between the two cases. Figure 11 illustrates a network that maps the most relevant Hallmark pathways to breast cancer with the corresponding GLMQL-MAS selected genes, demonstrating the interconnected nature of these biological pathways and their associated genes in the context of breast cancer.

## 3. Discussion

The application of the GLMQL-MAS methodology in this study has significantly advanced the analysis of gene expression data pertaining to ALNM in breast cancer. By enhancing data separability and highlighting key molecular markers, this approach has profound implications for understanding and managing breast cancer.

### 3.1. Applying GLMQL-MAS for the Analysis of Axillary Lymph Node Metastasis

Figure 1 illustrates the challenges of distinguishing ALNM+ from ALNM− samples using traditional logistic regression without the GLMQL-MAS filtering. The t-SNE projection and accompanying confusion matrix depict how unfiltered data can lead to substantial overlap between classes, emphasizing the necessity for robust data processing techniques to enhance data clarity and utility in clinical settings.

Figure 2 showcases the volcano plot that details the expression changes in genes between ALNM+ and ALNM− groups, identified as significant through the GLMQL-MAS methodology. The strict threshold of |LogFC| > 1 ensures focus on genes with substantial expression differences, highlighting the method’s efficacy in pinpointing biologically relevant markers. Figure 3 along with Figure S1 further illustrate the effectiveness of the GLMQL-MAS process using a refined subset of BH-significant genes. The t-SNE projections and logistic regression decision boundaries in this figure demonstrate improved separability and clearer classification of ALNM status, confirming the value of GLMQL-MAS in enhancing the interpretability of complex gene expression data.

Figure 4, along with Figures S2 and S3, explores the robustness of the GLMQL-MAS methodology across various thresholds of log fold change (LogFC). This demonstration shows that the system effectively identifies and prioritizes genes with both significant adjusted *p*-values and high LogFC, thus ensuring the identification of genes with the most pronounced biological impacts.

Figure 5 provides a compelling visualization of the effectiveness of the GLMQL-MAS methodology in refining gene expression data analysis for breast cancer. The enhancements in model performance metrics, specifically sensitivity and area under the curve (AUC), underscore the substantial impact of this gene filtering process on improving the discrimination capabilities of logistic regression models analyzing ALNM status.

The significant increase in sensitivity, from approximately 0.571 to 0.929, demonstrates that GLMQL-MAS not only identifies genes that are statistically significant but also ensures these genes are biologically relevant for distinguishing between ALNM+ and ALNM− samples. This substantial improvement suggests that the selected genes contribute meaningful insights into the biological differences between the two groups, enhancing the model’s accuracy in classifying the samples correctly.

Moreover, the corresponding rise in AUC highlights the overall effectiveness of the model across various decision thresholds, indicating a robust predictive performance facilitated by the refined gene selection. This metric confirms that the genes selected through GLMQL-MAS are not only enhancing sensitivity but also improving the overall reliability and interpretability of the model.

By leveraging the GLMQL-MAS process, researchers can achieve a deeper understanding of the molecular dynamics involved in lymph node metastasis, aiding in the development of more targeted diagnostic and therapeutic strategies. The results from Figure 5, therefore, not only validate the utility of GLMQL-MAS in research settings but also suggest its potential application in clinical practice, where precise molecular characterization is crucial for patient management.

Figure 6 along with Figure S6 demonstrate that the top-selected genes by the GLMQL-MAS system remain very stable even when we switch to random selection to balance the negative and positive samples. It turns out that some, such as the family of small proline-rich proteins (SPRR), are extremely stable during sampling and are consistently upregulated significantly.

Within the family of small proline-rich proteins (SPRR), *SPRR2B* and other members like *SPRR2E* are of particular interest in oncology due to their roles in cancer progression [40]. Yao et al. [41] demonstrated that higher *SPRR2B* levels in gastric cancer (GC) tissues correlate significantly with advanced tumor size, stage, and poorer survival, implicating *SPRR2B* in GC progression through the MDM2-p53/p21 pathway. Zhang et al. [42] identified *SPRR2E* as a key component in a prognostic cluster for oral squamous cell carcinoma (OSCC), linked to crucial pathways like the cornified envelope and peptide cross-linking, with high *SPRR2E* levels predicting worse survival in *OSCC* patients. Hao et al. [43] noted overexpression of *SPRR2D* in early-stage prostate cancer, suggesting its role in advancing cancer severity, while Liao et al. [44] found *FAM9C* to significantly influence breast cancer prognosis, linking it to key pathways affecting tumor progression. Cilek et al. [45] highlighted *FAM9C* as a significant player in miRNA-mediated mechanisms in trastuzumab-treated HER2+ breast cancer cells. Takan et al. [46] explored the functional implications of *KRT28* within cancer, noting its involvement in cell invasion and metastasis. Larson et al. [47] and Zhan et al. [48] established *SFTA3* as a diagnostic biomarker for lung adenocarcinoma, differentiating it from squamous cell carcinoma with high specificity. Hirata et al. [49] identified olfactory receptor *OR6C6* as a potential biomarker for predicting preeclampsia in gestational hypothyroidism. Wang et al. [50] revealed GADL1’s role in influencing pathways crucial to ovarian cancer progression. Fiegl et al. [51] associated higher NEUROD1 methylation levels with improved chemotherapy response and prognosis in breast cancer, while Ikematsu et al. [52] provided evidence of *NEUROD1*’s involvement in the aggressiveness of small cell lung cancer (SCLC), suggesting potential as a therapeutic target. Lastly, the discovery of *RXFP2*’s unique role in cancer, mediated by its interaction with INSL3, opens new avenues for targeted cancer therapies [53,54,55].

### 3.2. Comprehensive Gene Ontology (GO) and Gene Set Enrichment Analysis (GSEA) of Hallmark Gene Sets in Lymph Node Metastasis of Breast Cancer

The culmination of our analyses in Figure 10 and Figure 11 leverages a comprehensive suite of previous analytical frameworks, from Figure 7, Figure 8 and Figure 9 (and their corresponding figures in Supplementary Materials), to refine and emphasize the most robust and reliable results. These figures focus on the common gene ontology (GO) processes and Hallmark pathways influenced by GLMQL-MAS-selected genes across two comparative cases (Case 1, 65 ALNM− versus 42 ALNM+, and Case 2, random sampling of 42 ALNM− to compare against 42 ALNM+), showcasing their importance in understanding breast cancer metastasis. Since the genes highlighted are consistently significant across different comparisons, they represent some of the most reliable markers for ALNM and are potentially crucial for developing targeted therapies.

Figure 10 specifically delves into significant GO processes such as “nucleosome assembly” and “chromatin assembly or disassembly,” underscoring the role of chromatin remodeling in regulating gene expression critical for metastasis. These processes are pivotal for understanding the genetic mechanisms that facilitate or hinder the spread of cancer cells to lymph nodes. By identifying and focusing on these key biological processes, our study not only enhances understanding of metastatic progression but also aids in pinpointing potential targets for therapeutic intervention.

Moreover, the results from Figure 10 underscore the importance of these cellular mechanisms, which could potentially be targeted to disrupt the metastatic cascade at the molecular level. For example, therapeutic strategies might focus on inhibiting specific chromatin remodelers to halt the process of metastasis. Key genes such as *H3C10*, *H1-2*, *PADI4*, *H4C12*, *H3C2*, *H2BC17*, *H3C12*, *H4C3*, *H4-16*, *H2BU1*, *H2BC21*, *H4C4*, *H2BC6*, *H4C7*, *H1-3*, *H3C13*, *H4C2*, *H1-4*, and *H2BC18* appear across multiple GO terms, which are essential in DNA packaging, nucleosome assembly, and chromatin remodeling. These processes shape gene expression patterns influencing cellular behavior during cancer progression, suggesting their central role in these pathways and making them promising targets for drug development.

Chen et al. [56] demonstrated *H3C10*’s upregulation in colorectal neuroendocrine carcinomas (CRNECs) and its association with poor survival, suggesting its potential as a prognostic biomarker. Gu et al. [57] highlighted the role of H1-2 in promoting chemoresistance and epithelial-mesenchymal transition (EMT) in pancreatic cancer, mediated through the c-MYC signaling pathway, suggesting targeting H1-2 could improve therapeutic outcomes by reducing metastatic potential. Liu et al. [58] indicated PADI4’s involvement in lung cancer metastasis, with its reduction leading to decreased migratory and invasive capabilities, linked to EMT processes. Bonner et al. [59] reported significant somatic mutations in histone genes across various cancers, with mutations in H3C2 and H2BC6 particularly affecting histone function and chromatin remodeling. These mutations, prevalent in pediatric CNS tumors like diffuse midline glioma, are critical in cancer pathogenesis and development, highlighting the potential of histone-targeted therapies and enhancing our understanding of histone mutations in cancer.

Hannan et al. [60] identified crucial histone genes *H2BC21*, *H3C12*, *H2BC17*, *H3C2*, and *H3C10* through a protein–protein interaction (PPI) analysis, suggesting their significant role in chromatin remodeling and regulation in gastric cancer pathogenesis from data across three GEO datasets. Jafari et al. [61] highlighted histone-encoding genes *H4C* and *H1-4* as potential prognostic markers in pancreatic cancer by analyzing TCGA data, noting these genes among the top differentially expressed between cancerous and normal tissues, suggesting their role in chromatin dynamics could be targets for future therapies. Similarly, Jia et al.’s [62] study on the H2B gene family, particularly *H2BC21*, showed its high expression correlates with poor glioma prognosis and is involved in critical cancer pathways like cell cycle regulation and immune responses, using data from TCGA, CGGA, and GEO, indicating its potential as a biomarker for glioma progression.

Tang et al. [63] demonstrated that targeting *CD44* with anti-CD44s monoclonal antibody *H4C4* in pancreatic cancer significantly inhibits tumor growth, metastasis, and recurrence, with substantial in vitro and in vivo effects on cancer stem cells, highlighting its potential as a therapeutic agent. Espiritu et al. [64] investigated the *H4C7* histone isoform’s role in cancer, particularly noting its involvement in ribosomal DNA transcription and its correlation with breast cancer progression stages, suggesting its utility as a biomarker. Medrzycki et al. [65] studied the impact of overexpressing histone H1-3 in ovarian cancer cells, which led to reduced growth and altered gene expression by repressing the oncogene H19, proposing *H1-3* as both a biomarker and a potential therapeutic target due to its regulatory effects on chromatin structure and gene expression.

Rashid et al. [66] reported that *H3C13*, a variant of the *H3.2* histone gene, is overexpressed across a variety of human cancers, indicating its potential role in altering chromatin organization and gene expression, thus influencing cancer pathogenesis. Similarly, Huang et al. [67] found that the necroptosis-related gene *H2BC18* significantly impacts the progression and immune environment of colorectal cancer, with their developed prognostic model predicting CRC outcomes effectively, suggesting that targeting H2BC18 could improve immunotherapy precision and treatment customization.

The insights presented in Figure 11 and Figure S11 form the core of our analysis, highlighting significant Hallmark gene sets and their implications in the management and detection of ALNM in breast cancer.

Hallmark Apoptosis (Overlap Genes: *CCNA1*, *NEFH*, *ERBB2*): Apoptosis or programmed cell death is critical in cancer progression [68]. As shown in Figure 11 and Figure S11, critical genes within the Hallmark Apoptosis pathway, such as *ERBB2* (*HER2*) [69] and *CCNA1* [70] are crucial for regulating tumor aggressiveness and responsiveness to therapy. ERBB2’s prominent role is particularly well documented, serving as a marker for poor prognosis and as a therapeutic target [71,72,73,74]. This emphasizes the potential for targeted therapies that can inhibit *ERBB2*, possibly limiting the metastatic spread to lymph nodes.

Hallmark Emt (Epithelial-Mesenchymal Transition) (Overlap Genes: *SCG2*, *FOXC2*, *MEST*, *ELN*): EMT is a process where epithelial cells lose their cell polarity and adhesion, gaining migratory and invasive properties [75]. This hallmark is directly relevant to cancer metastasis, including the spread to lymph nodes. *FOXC2* (Forkhead Box C2) is a transcription factor that plays a crucial role in the regulation of embryonic development, tissue homeostasis, and cell differentiation. In the context of cancer, particularly breast cancer, *FOXC2* is significant for its involvement in several critical processes that promote tumor progression and metastasis [76,77,78]. Hollier et al. [76] has shown that the expression of *FOXC2* is associated with increased metastatic potential in breast cancer cells. Its expression correlates with a more aggressive phenotype and poorer prognosis, particularly due to its role in promoting EMT and subsequent metastasis to sites like the axillary lymph nodes. *FOXC2* influences several downstream targets and pathways that are key in cell adhesion, migration, and invasion [79].

Hallmark Tgf Beta Signaling (Overlap Genes: *LEFTY2*, *NOG*): TGF-β signaling plays a dual role in cancer, acting as a tumor suppressor and a promoter of tumor progression and metastasis in advanced stages [80]. TGF-beta signaling genes such as *LEFTY2* [81,82] identified in Figure S11, are key in the EMT process, facilitating tumor cell migration and invasion.

Hallmark Angiogenesis (Overlap Gene: VTN): Angiogenesis, the formation of new blood vessels, is crucial for tumor growth and metastasis [83]. *VTN* (vitronectin) is involved in cell adhesion and matrix remodeling, which are key in metastatic dissemination [84].

Hallmark Il6 Jak Stat3 Signaling (Overlap Genes: *INHBE*, *REG1A*): This signaling pathway is involved in inflammation and immune responses and has been linked to breast cancer progression and metastasis [85]. *REG1A* (Regenerating Family Member 1 Alpha) has been implicated in various cancers, including breast cancer [86]. In the realm of cancer biology, *REG1A* is particularly interesting due to its involvement in promoting cell growth, survival, and resistance to apoptosis, factors that are crucial for tumor progression and metastasis. Elevated levels of REG1A have been associated with poor prognosis in several types of cancers, as its expression can contribute to the aggressive behavior of cancer cells, including enhanced metastatic potential [87]. This makes REG1A a potential biomarker for identifying high-risk cancer patients and a possible target for therapeutic intervention, aiming to inhibit its cancer-promoting activities.

Hallmark Hypoxia (Overlap Genes: *BCAN*, *ALDOB*, *TKTL1*, *NCAN*, *ACKR3*): Hypoxia (low oxygen levels) in tumors triggers various mechanisms promoting survival, angiogenesis, and metastasis [88,89]. Genes such as *ACKR3* are associated with hypoxic responses and may contribute to the lymphatic spread of tumor cells [90].

Hallmark Kras Signaling UP and Hallmark *KRAS* Signaling DN (Overlap Genes including *PTGS2*, *IGF2*): *KRAS* signaling is pivotal in many cancers, affecting cell growth, apoptosis, and migration [91]. These pathways could indirectly influence lymph node metastasis. The expression of PTGS2 in tumor tissues is linked to several key aspects of cancer progression [92,93,94,95]. It promotes tumor growth [96], angiogenesis [97], and metastasis [98].

## 4. Materials and Methods

For our study, we harnessed the extensive dataset from the Cancer Genome Atlas (TCGA) Breast Invasive Carcinoma (BRCA) cohort [99], which comprises genomic and clinical data from 1078 patients. This dataset provides a solid foundation for investigating the molecular traits of breast cancer and its clinical outcomes. To reduce confounding variables and precisely evaluate the genetic landscape linked to lymphatic spread, we specifically selected patients who had not undergone chemotherapy or radiotherapy prior to sampling; from these, only subjects with ALNM RNA-Seq data were included from the TCGA-BRCA.

**Figure 12:**
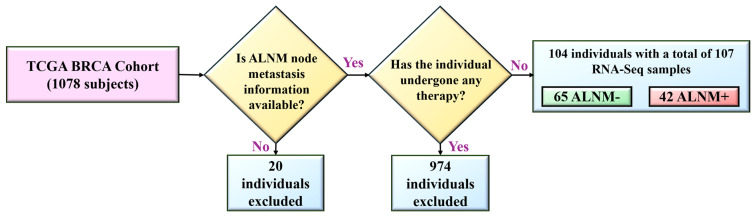
The flowchart details the criteria used to refine the initial dataset of 1078 patients down to 104 untreated individuals based on the availability of ALNM information and absence of prior treatment. The final cohort is categorized into two groups: 65 without ALNM (ALNM−) and 42 with ALNM (ALNM+), facilitating the study of genetic and molecular markers associated with lymph node metastasis.

Figure 12 illustrates the subject selection process within our study cohort through a detailed flowchart. Initially, we excluded 20 individuals lacking ALNM information. We then further refined our cohort by omitting any patients who had received chemotherapy or radiotherapy, narrowing it down to 104 untreated individuals. These were divided into two groups based on their RNA-Seq ALNM status: 65 without ALNM (ALNM−) and 42 with ALNM (ALNM+). This careful selection ensured a focus on a naturally occurring tumor environment, ideal for examining the biological and molecular dynamics underlying lymph node metastasis.

Focusing on patients who had not received prior chemotherapy or radiotherapy is crucial for several reasons. First, it ensures an untainted genetic profile, as these treatments can induce significant genetic and molecular changes in tumor cells. By selecting patients who have not undergone these treatments, we ensure that the genetic data we analyze reflects the natural tumor environment without the confounding effects of treatment-induced alterations, providing a clearer picture of the genetic determinants and mechanisms driving lymph node metastasis in its untreated state. Second, treatment interventions can complicate the interpretation of genetic data due to the induction of secondary genetic alterations and selective pressures that may not be inherently linked to the cancer’s natural progression [100]. Studying the untreated phenotype allows for more straightforward associations between specific genetic markers and disease outcomes, enhancing the reliability of our findings. Additionally, by analyzing the genetic profiles of untreated patients, we establish a baseline understanding of the molecular drivers of ALNM. This provides a foundation for comparing how treatments might alter the genomic landscape, potentially influencing the pathways involved in metastatic spread and resistance mechanisms. Ultimately, by concentrating on untreated patients, our study aims to derive insights that are directly applicable to early intervention strategies and the development of targeted therapies that can be implemented at the initial stages of breast cancer management, potentially before conventional treatments begin.

The classification of lymph node involvement (ranging from $N_{0}$ to $N_{3}$) is vital for staging breast cancer and guiding therapeutic strategies, reflecting the disease’s spread and prognosis [101,102]. A meta-analysis encompassing 58 studies highlighted that even micro-metastases in axillary lymph nodes significantly elevate the mortality risk compared to patients without nodal involvement [103].

From the TCGA database portal, we downloaded RNA-Seq Transcriptome Profiling Gene Counting data for the 104 patients. The data include a comprehensive count of 60,660 genes, including types such as protein-coding, lncRNA (long non-coding RNA), miRNA (microRNA), and pseudogenes. The bioinformatics processing of the RNA-Seq data was performed using the GDC mRNA quantification analysis pipeline, which begins with the STAR algorithm [104] to align reads to the GRCh38 reference genome and generate raw read counts [105].

Given the crucial role of protein-coding genes in cellular processes and disease mechanisms, we focused our analysis on these genes. Protein-coding genes are directly involved in cellular pathways and are more likely to affect disease phenotypes through mutations that alter protein function [106]. Therefore, our dataset specifically emphasizes the analysis of 19,938 protein-coding genes per RNA-Seq sample, enabling us to explore their potential as predictive markers for lymphatic metastasis in breast cancer.

### 4.1. Generalized Linear Models with Quasi-Likelihood F-Tests and Magnitude-Altitude Scoring (GLMQL-MAS)

In this section, the primary objective is to conduct a comprehensive expression analysis to elucidate the differential gene expression associated with lymph node metastasis in breast cancer. This analysis is pivotal for identifying key genes and molecular pathways that may play a significant role in the progression and spread of breast cancer to lymph nodes.

Our approach to expression analysis is methodologically robust, incorporating state-of-the-art statistical techniques and normalization methods to ensure the reliability and accuracy of our findings. We define stringent criteria for statistical significance based on adjusted *p*-values to account for multiple testing, ensuring that differentially expressed genes (DEGs) are identified with high confidence. Observations with adjusted *p*-values (Benjamini Hochberg [107,108] or Bonferroni [109] corrections) smaller than α=0.05 are deemed significant, highlighting potential DEGs.

In processing and analyzing our RNA-Seq dataset, we begin with a crucial preprocessing step, TMM normalization, facilitated by the EdgeR package [27]. This normalization is pivotal for adjusting differences in the library size and compositional variances across our samples, laying the groundwork for a precise comparison of gene expression across the different experimental groups, namely those with and without lymph node metastasis. Organizing the count data into a DGEList object correlates each gene count with its respective experimental condition, whether ALNM− or ALNM+.

RNA-Seq data introduce distinct challenges for analysis due to their inherent non-normal distribution and common occurrence of overdispersion, where the variance of the data exceeds the mean [110]. Traditional parametric testing methods, such as the *t*-test, are unsuitable for RNA-Seq data analysis due to these characteristics. Our study employs generalized linear models (GLMs) [111] for the statistical modeling of RNA-Seq count data, leveraging functions within the EdgeR [27]. GLMs are adept at accommodating error distribution models beyond the normal distribution, ideal for count data that follow distributions from the exponential family, such as Poisson or negative binomial distributions. This is particularly relevant for RNA-Seq data, given its discrete nature and propensity for overdispersion.

Further, our analysis is enhanced by implementing the Quasi-Likelihood F-test [112] within the GLM framework. This test evaluates gene expression differences between groups without the stringent assumptions required by traditional parametric tests, enabling more precise and reliable statistical inference. Our application of GLMs alongside the Quasi-Likelihood F-test, designed to identify differentially expressed genes accurately, considers the unique distributional characteristics and gene-specific variability inherent in RNA-Seq data.

The steps involved in the GLMs with the Quasi-Likelihood F-tests process are detailed as follows:

1. Formation of Design Matrix:
  - A design matrix is constructed to reflect the experimental design, encapsulating all factors believed to influence gene expression (model.matrix()).
  - It organizes samples in rows and experimental factors in columns, facilitating the modeling of gene expression influences.
2. GLM Fitting:
  - The TMM-normalized count data are subjected to a GLM fitting process, utilizing a link function appropriate for count data, typically the log link (glmQLFit()).
  - The GLM quantifies the expected count values’ relationship to the linear predictors, with coefficients indicating log fold changes (LogFC) for each experimental factor.
3. Application of Quasi-Likelihood F-test:
  - Post model fitting, the Quasi-Likelihood F-test compares the full model against a reduced model to evaluate the impact of specific factors on gene expression (glmQLFTest()).
  - It directly estimates data dispersion, addressing the overdispersion characteristic of RNA-Seq data.
  - The test produces *p*-values to assess the significance of expression differences attributed to the experimental factors.
4. Extraction of Significance and Log Fold Change (LogFC):
  - Genes showing significant model deviations yield *p*-values.
  - Concurrently, GLM calculates LogFC values, reflecting the magnitude of expression change between treatment and reference conditions.

Our analytical rigor is fortified by a dual-layered approach to statistical validation, incorporating both the Benjamini–Hochberg (BH) [107,108] and Bonferroni corrections [109]. Advancing our analytical exploration, we employ the MAS into our methodological repertoire [31].

**MAS Definition:** At the core of MAS is the integration of two critical dimensions: expression change magnitude and statistical robustness. Specifically, for each gene of interest, MAS computes a score by merging the absolute ${log}_{2}$ fold change (${|{log}_{2}(\mathrm{FC}}_{l})|$) with the negative ${log}_{10}$ of its BH-adjusted *p*-value ($\left|{{log}_{10}(p}_{l}^{BH})\right|$). For each BH-significant gene $g_{l}$, this composite score represented as ${MAS}_{l}={|{log}_{2}(\mathrm{FC}}_{l}{)|}^{M}|{{log}_{10}(p}_{l}^{BH}){|}^{A}$, where $p_{l}^{BH}$ denotes BH-adjusted *p*-values. Here, M and A serve as adjustable hyperparameters that fine-tune the balance between the magnitude of expression change and its statistical significance, enabling a customized evaluation of each gene’s importance. By assigning a value of 1 to both M and A, we affirm that both the extent of expression change and its statistical validation hold equivalent weight in the overall scoring algorithm.

Incorporating the MAS into our methodology presents several advantages over traditional gene prioritization methods that rely solely on *p*-values or log fold changes (LogFC), as typically employed by analytical tools like EdgeR [27] or DESeq2 [113]. The MAS framework enhances gene significance evaluation by integrating the magnitude of expression changes with their statistical reliability, allowing for the identification of genes that signify both meaningful biological changes and statistical significance, a nuance often missed by singular metrics like *p*-values (or adjusted *p*-value) or LogFC. This approach recognizes that a gene’s impacts on disease processes stems not just from statistical significance but also from the biological magnitude of its expression changes. Additionally, the flexibility of MAS allows for the adjustment of hyperparameters M and A, tailoring the analysis to align with the specific objectives of our study and enabling a focus on either the magnitude of change or its statistical significance, providing a custom-fit analytical approach beyond the capabilities of more conventional methods. In this study, both M and A are set to 1.

**Validating MAS Ranking’s Power:** To demonstrate the MAS ranking system’s efficacy, we compared the top-selected genes following three levels of significance criteria (Significant (raw *p*-values), BH-Significant, and Bonferroni-Significant) as we varied the absolute value of the Log Fold Change (LogFC) from 0 to 7. This comparison aimed to validate the MAS ranking’s consistency and its independence from the LogFC threshold.

Furthermore, we applied a comprehensive analytic approach to differentiate between ALNM+ and ALNM− samples, utilizing principal components [114] to effectively reduce the dimensionality of our dataset. Specifically, we first transformed the entire gene expression data, as well as the subset of genes selected by our GLMQL-MAS methodology, into a reduced number of principal components. This reduction was executed to capture the major variance within the data while minimizing information loss, focusing on the most impactful genetic features.

Using these principal components, we then conducted logistic regression analysis [115] to determine how well the selected genes (after GLMQL-MAS was applied), versus the entire gene dataset (before GLMQL-MAS was applied), could distinguish between negative and positive ALNM samples. We gradually increased the number of principal components used in the analysis from 2 to 20. This increment allowed us to evaluate the impact of adding more genetic information (in the form of principal components) on the model’s ability to accurately classify the samples.

In this context, sensitivity refers to the model’s ability to correctly identify ALNM+ samples, effectively measuring the true positive rate. Conversely, specificity assesses the model’s capability to correctly exclude ALNM− samples, indicating the true negative rate. These metrics are crucial as they directly reflect the effectiveness of our selected genetic markers in accurately identifying the presence or absence of lymph node metastasis, a key factor in breast cancer prognosis and treatment planning.

Note that this analysis was not aimed at developing predictive models for clinical use but rather at demonstrating the robust capabilities of the GLMQL-MAS approach in selecting highly relevant genes. By comparing the sensitivity and specificity achieved with the principal components derived from the selected genes versus those derived from the entire gene set, we could directly assess the precision with which the GLMQL-MAS-selected genes pinpoint the genetic underpinnings of ALNM.

### 4.2. Applying GLMQL-MAS for the Analysis of Axillary Lymph Node Metastasis

In our study, the GLMQL-MAS ranking system serves as a foundational framework for deciphering differential gene expression linked to lymph node metastasis in breast cancer. This novel approach marries the statistical rigor of GLMQL for uncovering genes with significant expression differences with the MAS methodology for ranking these genes based on both their biological significance and statistical robustness.

#### Differential Expression Analysis

**Case 1. 65 ALNM− Versus 42 ALNM+:**

Initially, our differential expression analysis utilizes GLMQL to compare 42 ALNM+ against the entire 65 ALNM− samples. This phase is crucial for isolating genes that exhibit Benjamini–Hochberg (BH) significant expression differences, setting the stage for more detailed examination.

**Case 2. Random Sampling of 42 ALNM− to Compare Against 42 ALNM+:**

We introduce a balanced sampling strategy to validate the robustness of our GLMQL-MAS framework and the significance of the identified genes. We randomly select 42 samples from the ALNM− cohort to form a control group, directly mirroring the ALNM+ group’s size. This balanced comparison is designed to enhance the reliability of our findings. By applying the GLMQL-MAS system to compare the ALNM+ group against a randomly selected ALNM− control group in 500 iterations, we rigorously test our results’ stability across diverse sampling scenarios.

To ensure the utmost reliability in identifying key genes, our methodology includes a critical stipulation: a gene must manifest as BH-significant (Benjamini–Hochberg significant) in at least 50% (250 out of 500) of the comparisons in the same directional trend: upregulation or downregulation. This stringent criterion is set to eliminate any genes that show mixed expression trends across the sampling iterations. Distinctly, we categorize potential biomarkers as either unequivocally BH-significant upregulated or downregulated, avoiding any overlap between these classifications. The strongest biomarker candidates are those genes that exhibit 100% consistency (500 out of 500 times) in their upregulation or downregulation patterns, indicating a robust association with lymph node metastasis. To further refine our analysis, we incorporate a significant biological dimension by focusing on genes with notable Log Fold Changes (LogFC). We establish initial thresholds of LogFC>1 for upregulated genes and LogFC<−1 for downregulated genes. Subsequently, we progressively increase these thresholds up to LogFC>6 and LogFC<−6, respectively. This strategy enables us to prioritize genes that are not only statistically significant but also possess considerable biological implications.

In summary, the study initially employs GLMQL to identify BH-significant genes through a comparison of ALNM+ versus ALNM− samples. Following this, MAS is applied to rank these genes, with a keen focus on their potential as key biomarkers. The subsequent sampling strategy, applied to balance the dataset, is designed to verify if the top genes identified through GLMQL-MAS in the full dataset analysis remain consistent across sampling iterations, underlining their potential as biomarkers. This integrated approach underscores our commitment to identifying key genes that play a pivotal role in the context of lymph node metastasis in breast cancer.

### 4.3. Comprehensive Gene Ontology (GO) and Gene Set Enrichment Analysis (GSEA) of Hallmark Gene Sets in Lymph Node Metastasis of Breast Cancer

Following the identification of significant genes involved in the lymph node metastasis of breast cancer, particularly those designated as BH-significant through our precise GLMQL-MAS ranking system, we proceeded to conduct a gene ontology (GO) analysis. This analysis utilized the clusterProfiler [116] and org.Hs.eg.db [117] packages within R, serving a pivotal role in categorizing the identified genes into groups associated with biological processes (BPs), cellular components (CCs), and molecular functions (MFs). By mapping our significant genes to specific GO terms, we were able to illuminate the functional characteristics of these genes, thereby providing a detailed view of their roles within the cellular environment. This comprehensive GO analysis not only underscored the biological processes most disrupted by lymph node metastasis but also pinpointed potential molecular targets for therapeutic intervention.

Building on the insights gained from the GO analysis, we further enhanced our understanding of the biological implications of our findings by conducting a gene set enrichment analysis (GSEA) using the Hallmark gene sets [36,37]. The Hallmark gene sets, curated to summarize and represent well-defined biological states or processes, provided a robust framework for interpreting the potential biological impact of the genes identified as significant in our study.

## 5. Conclusions

In this study, we aimed to advance the detection and management of axillary lymph node metastasis (ALNM) in breast cancer by integrating generalized linear models with quasi-likelihood (GLMQL) and magnitude altitude dcoring (MAS). Recognizing the limitations of traditional histopathological assessments of ALNM, which often delay treatment initiation, lead to significant morbidity, and require extensive expertise, we leveraged artificial intelligence and machine learning technologies in conjunction with RNA sequencing (RNA-Seq) to enhance the precision and efficiency of ALNM diagnostics.

Our methodology involved an exhaustive differential gene expression analysis, sifting through RNA-Seq data from the TCGA Breast Invasive Carcinoma (BRCA) cohort to identify 986 genes significantly associated with ALNM. This approach highlighted key biological processes through gene ontology (GO) analysis, such as nucleosome assembly, chromatin organization, and DNA packaging, providing a deeper understanding of the molecular mechanisms driving ALNM and opening new avenues for targeted therapeutic interventions.

The GLMQL-MAS methodology has identified a number of genes significantly associated with ALNM in breast cancer, providing critical insights into the molecular mechanisms driving cancer progression and metastasis. Among these genes are ERBB2, CCNA1, FOXC2, LEFTY2, VTN, ACKR3, and PTGS2, which influence processes such as apoptosis, epithelial–mesenchymal transition, angiogenesis, hypoxia responses, and KRAS signaling pathways, all vital for tumor aggressiveness and metastatic spread. Additionally, the GLMQL-MAS system highlighted the small proline-rich protein family (SPRR) including SPRR2B, SPRR2E, and SPRR2D, noted for their significant roles in cancer-related pathways and their potential as therapeutic targets. Key transcripts such as H3C10, H1-2, PADI4, and others were identified as crucial in regulating the chromatin architecture and gene expression, which are essential for cancer progression and metastasis.

### Limitations of Study

This study, while providing valuable insights into the detection and management of ALNM in breast cancer, encounters several limitations that warrant consideration:

1. The limited sample size significantly constrains the statistical power of our analyses, stemming from a focus on untreated patients within the TCGA Breast Invasive Carcinoma (BRCA) cohort. This decision was made to avoid the confounding effects of prior therapies, which could influence gene expression profiles.
2. The reliance on a predefined dataset from untreated patients limits the diversity of the patient population and does not reflect the typical clinical scenario where patients may receive neoadjuvant therapies. These selection criteria restrict our findings’ generalizability to all breast cancer patients.
3. The demographic and geographic homogeneity of the TCGA cohort might introduce selection bias, potentially influencing the study’s conclusions regarding populations that differ in ethnicity, age, or treatment history.

## Data Availability

The data used for our analysis are sourced from the publicly accessible Breast Invasive Carcinoma (BRCA) cohort of The Cancer Genome Atlas (TCGA), available through the National Cancer Institute’s Genomic Data Commons (GDC) Data Portal at https://gdc.cancer.gov/ (accessed on 10 January 2024). Access to the TCGA BRCA dataset requires registration with the GDC Data Portal and adherence to the data access guidelines specified by the TCGA program. This dataset is freely available for research purposes, within the confines of the data access policies and restrictions established by the TCGA program and its affiliated institutions. Further details on data access and usage policies are available on the TCGA and GDC websites.

## Supplementary Materials

The following supporting information can be downloaded at: https://www.mdpi.com/article/10.3390/ijms25137306/s1.
