# Supplementary material for: Identifying Key Genes Involved in Axillary Lymph Node Metastasis in Breast Cancer Using Advanced RNA-Seq Analysis: A Methodological Approach with GLMQL and MAS
Source: Int J Mol Sci. 2024 Jul 3;25(13):7306. doi: 10.3390/ijms25137306 (PMC11242629; doi:10.3390/ijms25137306)
Supplement: Supplementary file 1 [file ijms-25-07306-s001.zip › ijms-3057907-supplementary.pdf]

# Supplementary Material (SM) for

# Identifying Key Genes Involved in Axillary Lymph Node Metastasis in Breast Cancer Using Advanced RNA-Seq Analysis: A Methodological Approach with GLMQL and MAS

**Mostafa Rezapour [1,*], Robert Wesolowski [2] and Metin Nafi Gurcan [1]**

[1] Center for Artificial Intelligence Research, Wake Forest University School of Medicine, Winston-Salem, NC 27101, USA; mgurcan@wakehealth.edu

[2] Division of Medical Oncology, James Cancer Hospital and the Ohio State University Comprehensive Cancer Center, Columbus, OH 43210, USA; robert.wesolowski@osumc.edu

* Correspondence: mrezapou@wakehealth.edu

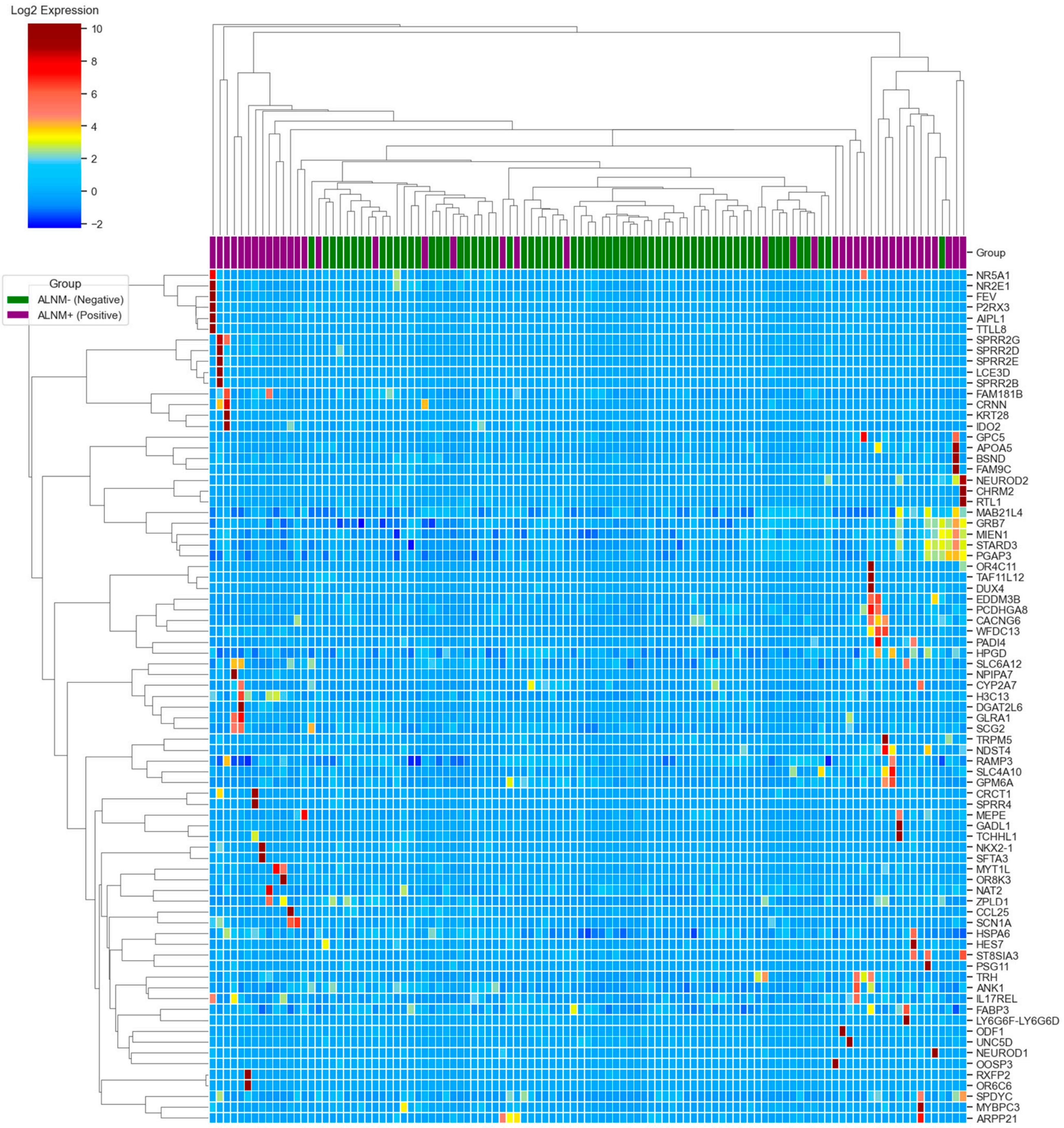


**Figure S1.** Hierarchical Clustering Heatmap of Gene Expression in Breast Cancer Samples. This figure illustrates the differential expression patterns of the top 100 genes selected using the GLMQL-MAS approach, filtered with a LogFC threshold of >1. Samples are grouped into ALNM- (negative for axillary lymph node metastasis, green bar) and ALNM+ (positive for axillary lymph node metastasis, purple bar), showcasing distinct molecular signatures that differentiate metastatic from non-metastatic status. Each row represents a gene, and each column a sample, with color intensity reflecting the log2 expression level as indicated by the color scale (blue: low expression, red: high expression).

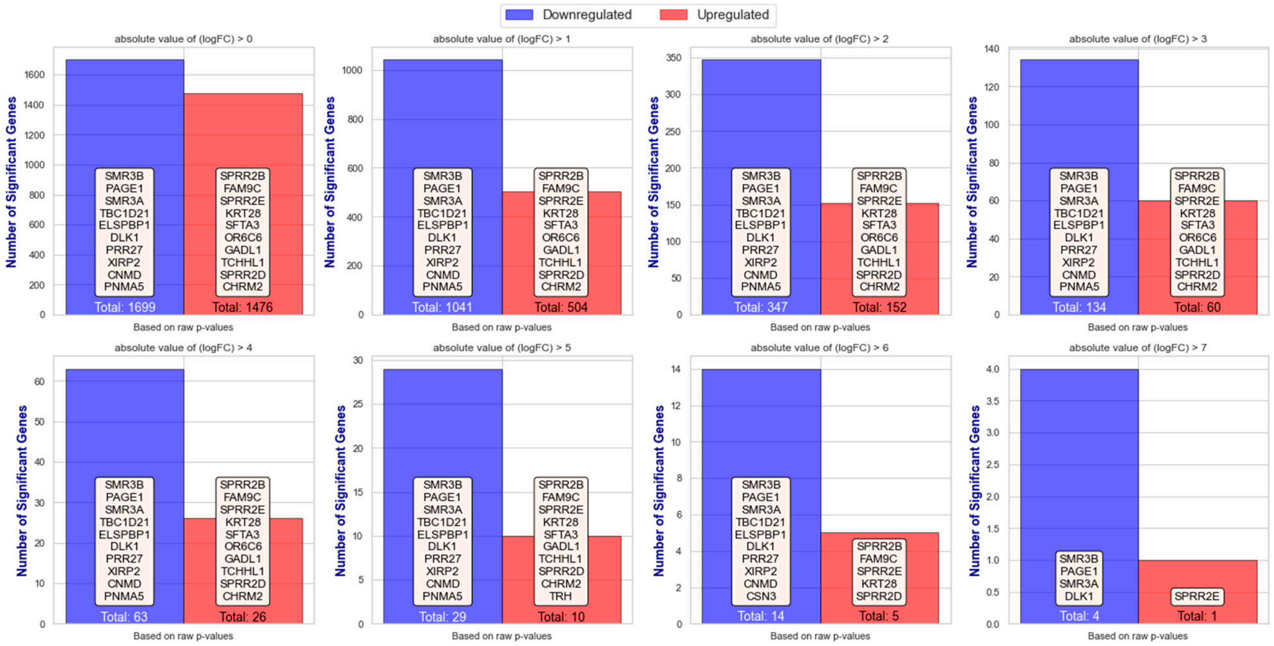


**Figure S2.** Stability of GLMQL-RMAS When Applying RMAS to Raw p-Values. This figure illustrates the consistent performance of the GLMQL-RMAS methodology in identifying significant genes, demonstrating its robustness by applying the RMAS to raw p-values, a testament to the method's reliability across different statistical treatments.

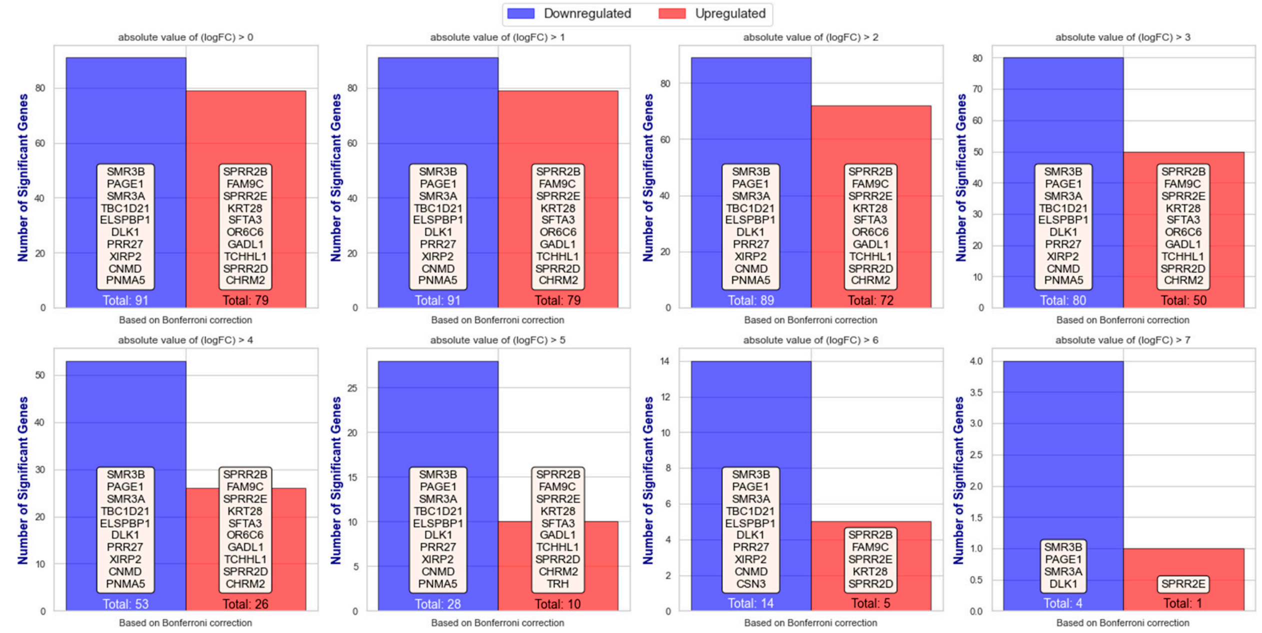


**Figure S3.** Effectiveness of GLMQL-MAS with Bonferroni Correction Method. This figure illustrates the performance of the GLMQL-MAS when implementing a Bonferroni correction rather than the Benjamini-Hochberg approach.

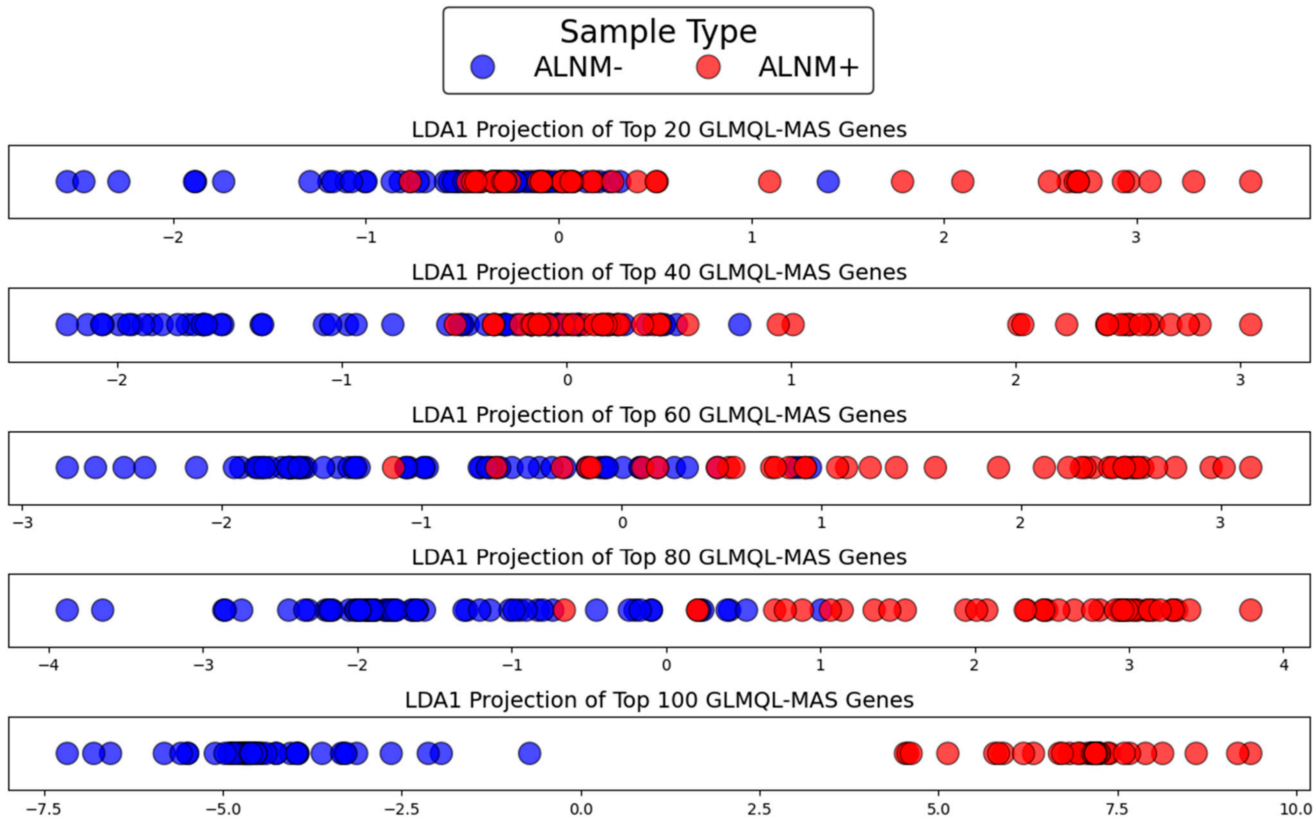


**Figure S4.** LDA1 projection illustrating the separation of ALNM+ and ALNM- samples using the top-n GLMQL-MAS selected genes, with n ranging from 20 to 100. Perfect separation (100%) is achieved at n=100, highlighting the discriminative power of the selected genes in distinguishing between the two groups.

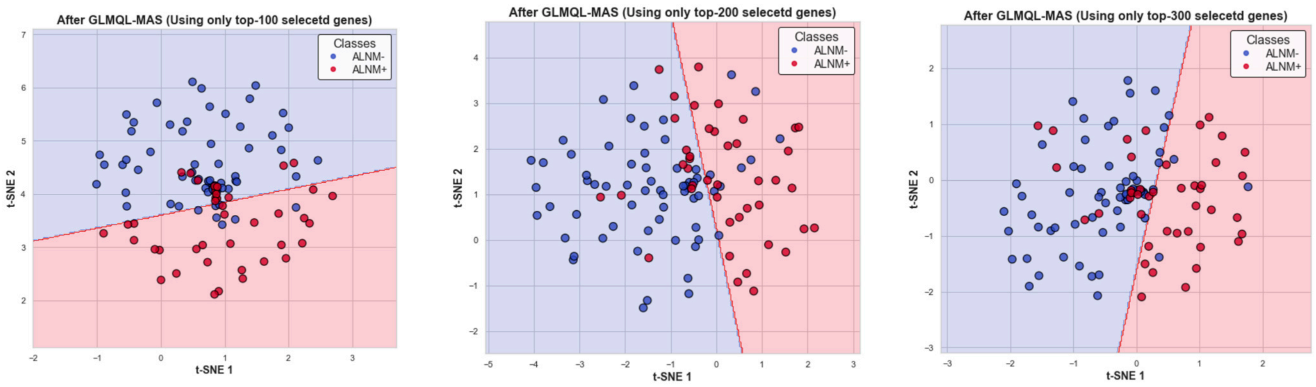


**Figure S5.** Logistic regression decision boundaries depicted using TSNE1 and TSNE2 coordinates for samples projected with top-n GLMQL-MAS selected genes. From left to right, the panels represent n=100, n=200, and n=300, showcasing the progressive improvement in differentiating ALNM+ from ALNM- samples through the GLMQL-MAS gene selection process.

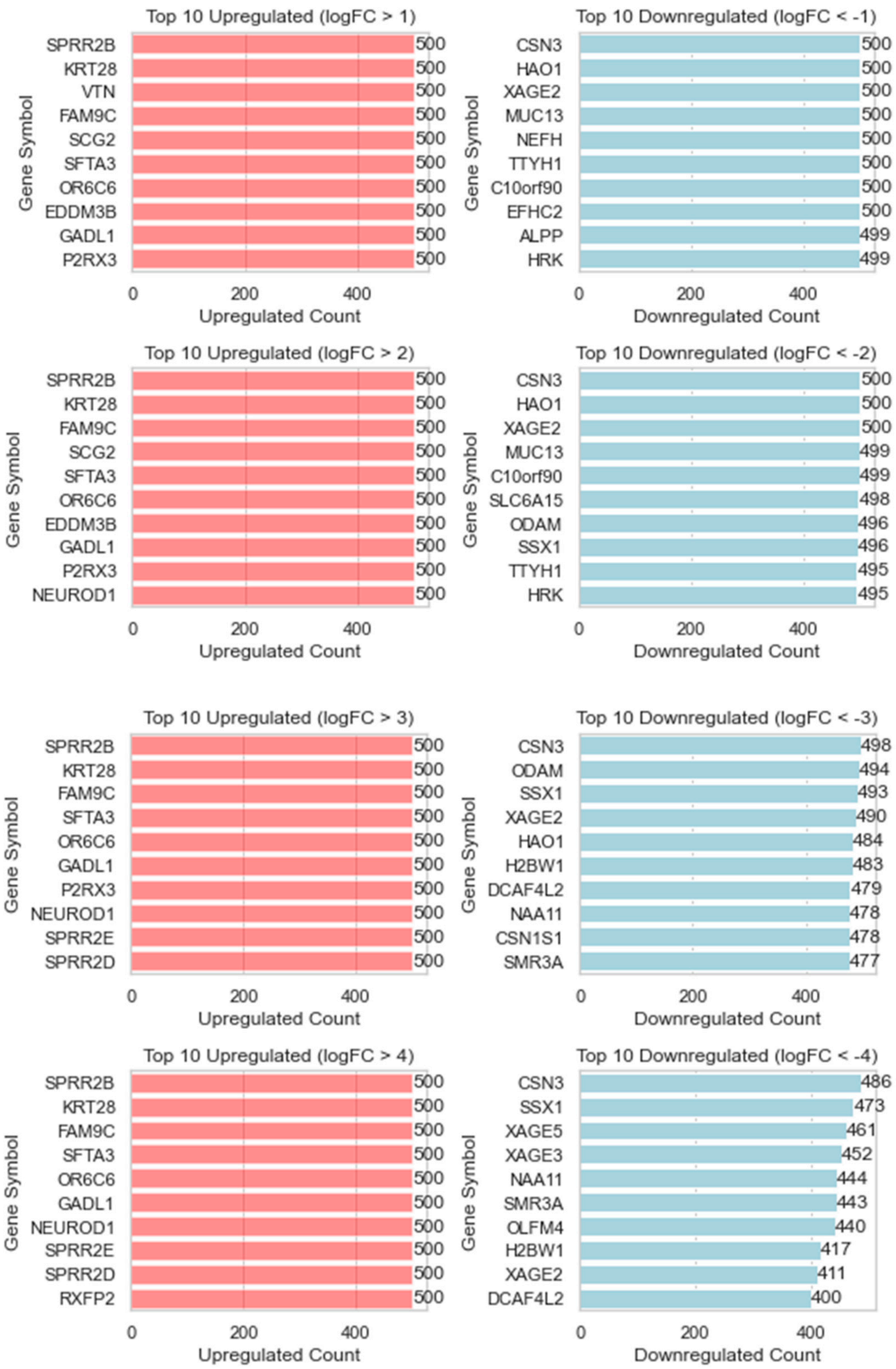


**Figure S6.** Distribution of the Top 10 Genes by BH-Significance Occurrence Using GLMQL-MAS. This figure illustrates the top-10 genes that achieved the highest frequency of BH-significance in an analysis of 500 iterations, with |LogFC| upper thresholds set at 1, 2, 3, and 4. It highlights the genes that consistently demonstrate significant differential expression in lymph node metastasis of breast cancer.

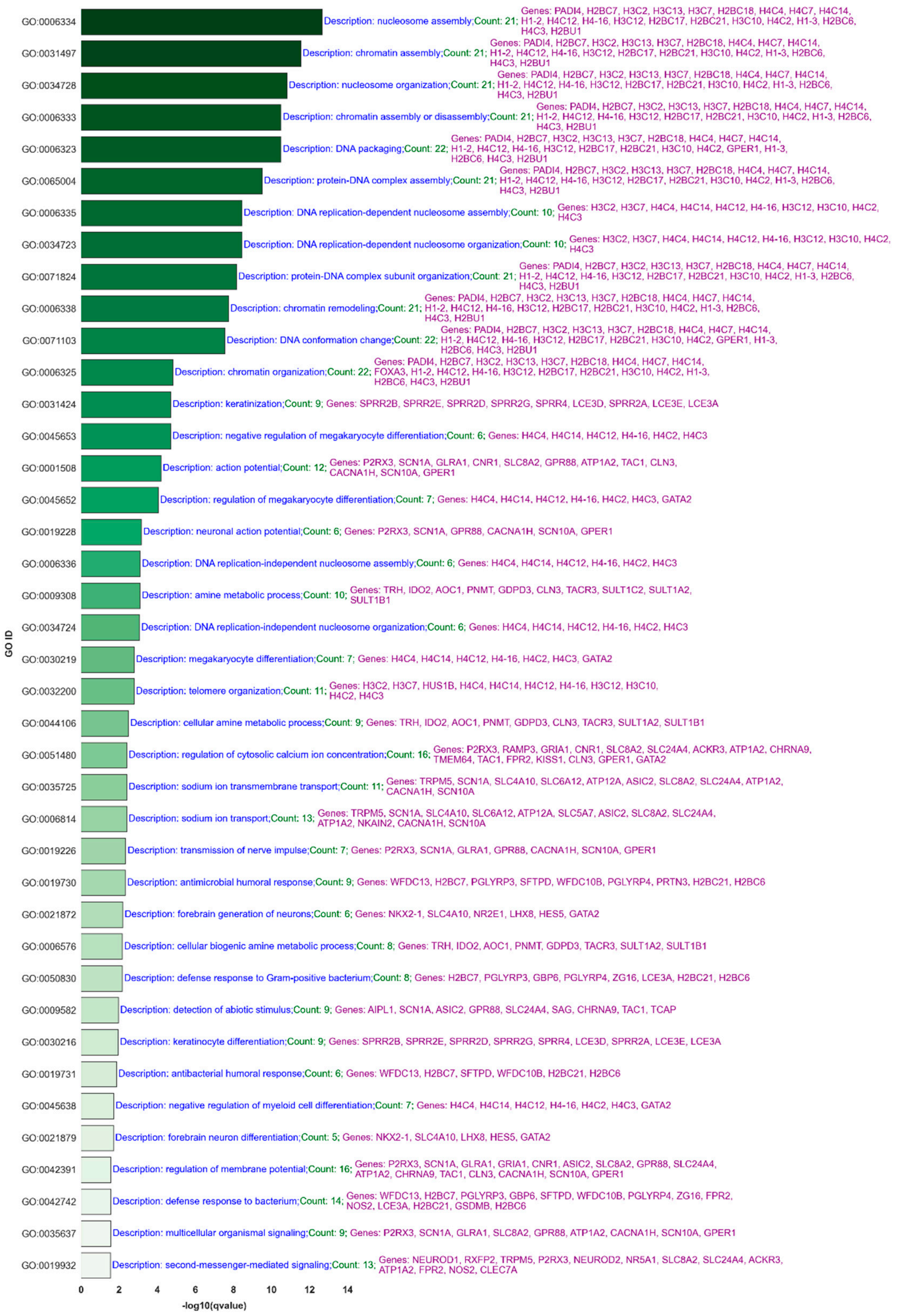


**Figure S7.** Top 10 GO Processes Related to Lymph Node Metastasis. This figure details the GO processes most intimately connected with the pathology of lymph node metastasis, providing insights into the molecular functions and cellular components affected.

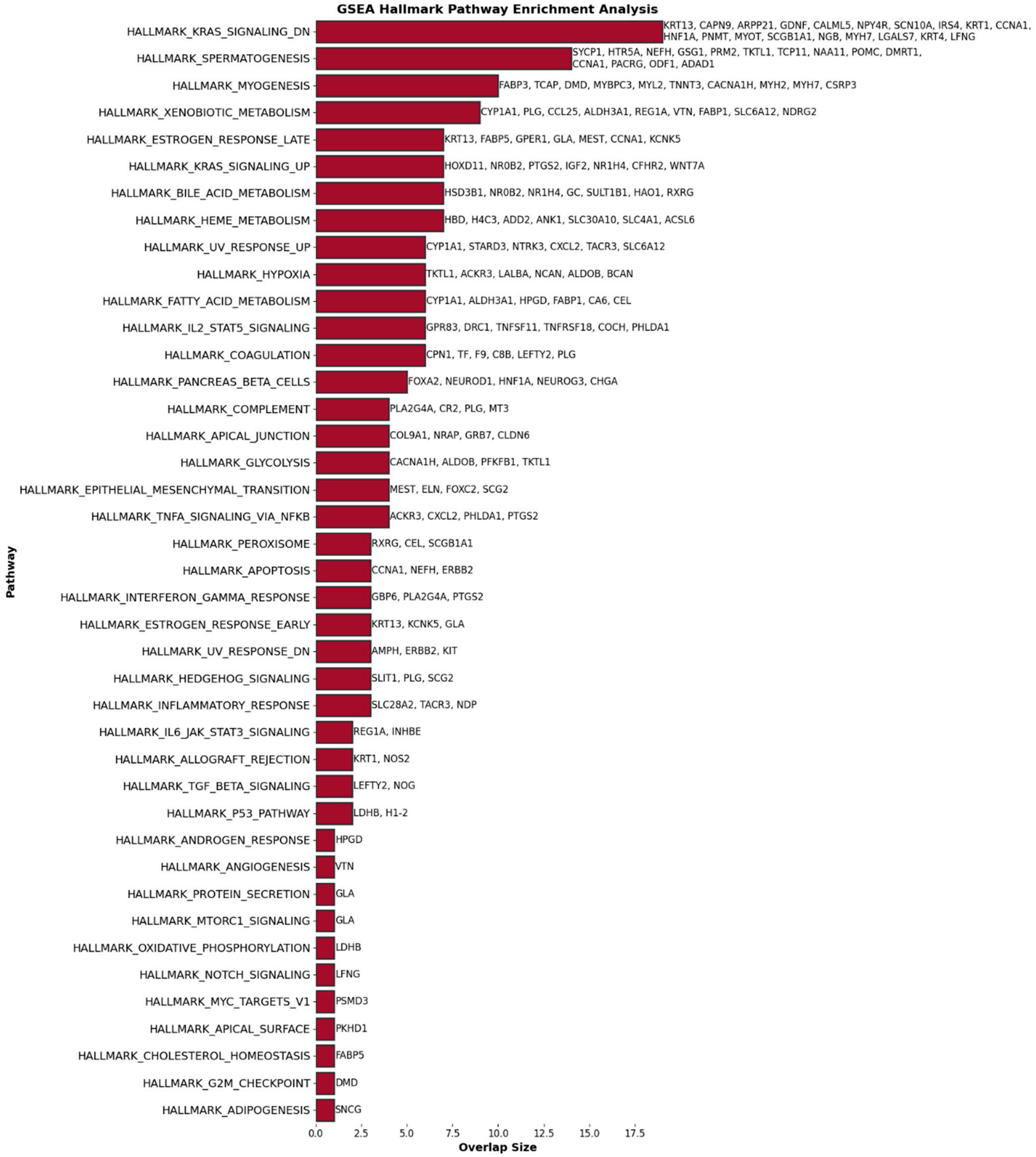


**Figure S8.** This figure illustrates the significant pathways identified from the GSEA using Hallmark gene sets, highlighting the predominant biological mechanisms influenced by the GLMQL-MAS selected genes between 42 ALNM positive (ALNM+) and 65 ALNM negative (ALNM-) breast cancer samples.

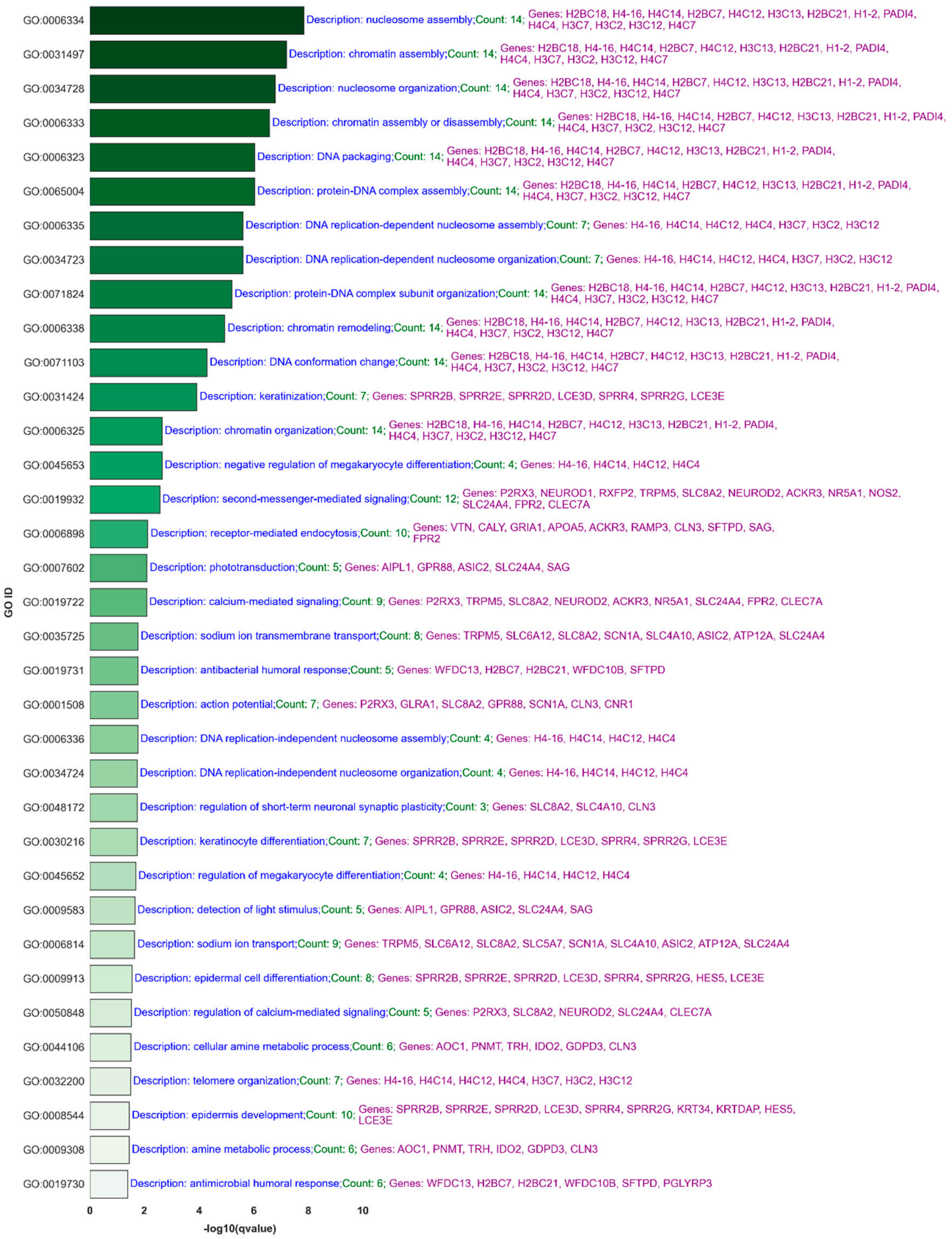


**Figure S9.** Significant GO Processes for Upregulated BH-Significant Genes. Showcasing the top q-value-based GO processes for genes identified as BH-significant and upregulated in at least 250 of 500 iterations. This visualization emphasizes the biological processes most consistently associated with lymph node metastasis.

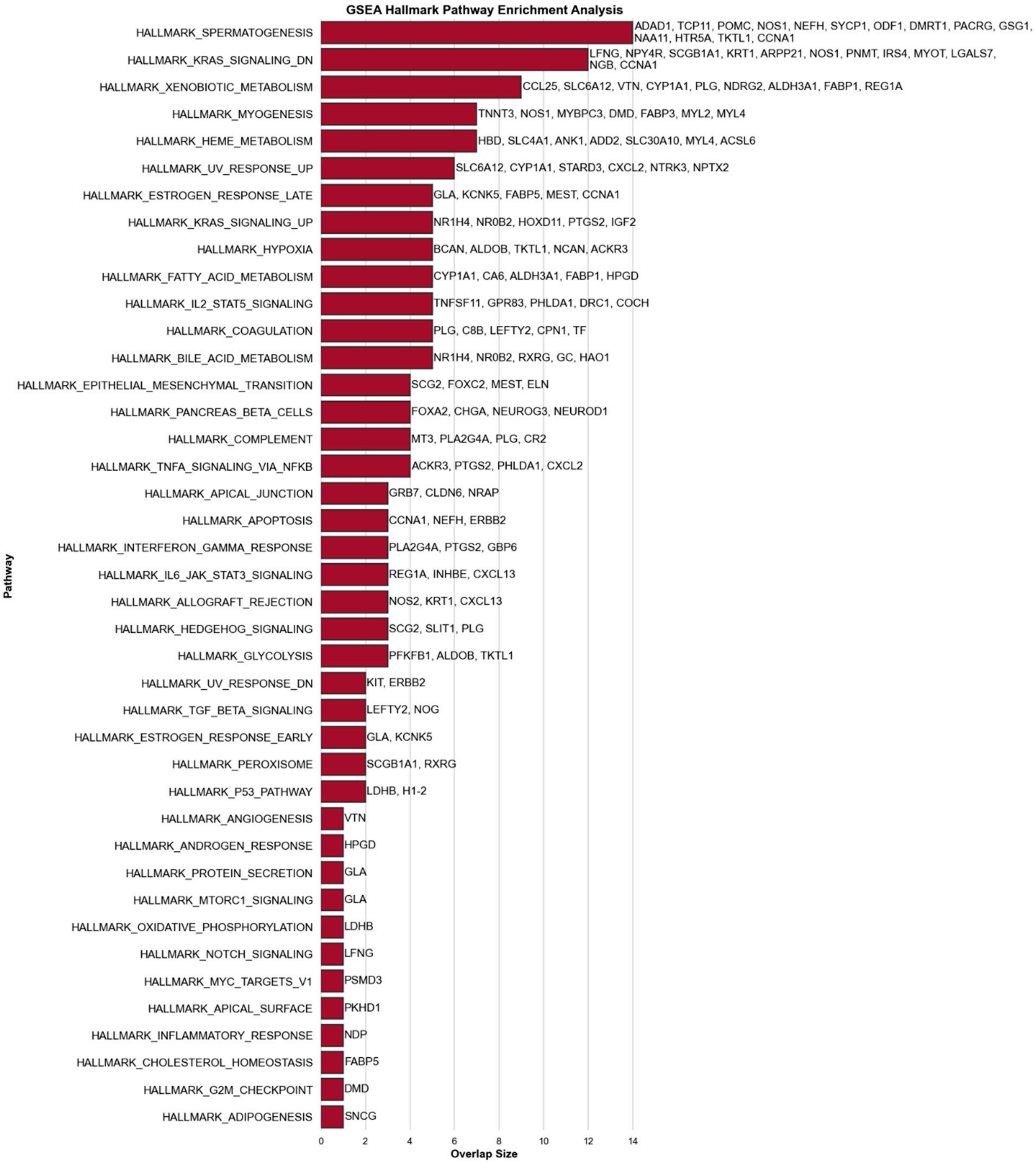


**Figure S10.** Gene Set Enrichment Analysis (GSEA) of Hallmark Gene Sets. This figure illustrates the enrichment results for Hallmark gene sets in the context of lymph node metastasis. Each bar represents a specific pathway, highlighting the pathways most significantly associated with the genetic changes observed in the ALNM+ group compared to the ALNM- group, based on q-values. This analysis underscores the biological relevance of the identified genes and their roles in critical cancer-related pathways.

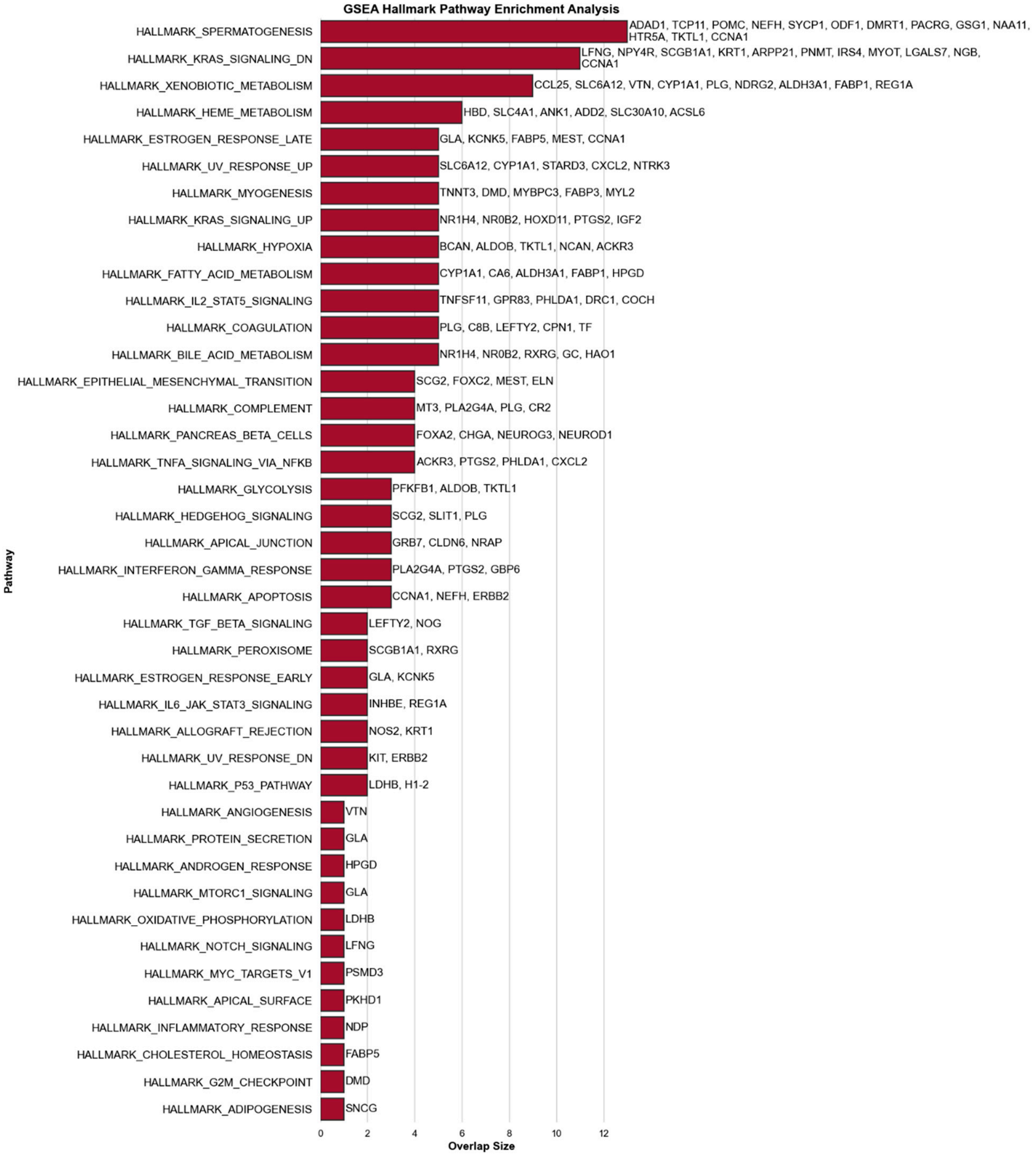


**Figure S11. Gene Set Enrichment Analysis (GSEA) Using Hallmark Gene Sets for Common Genes Across Two Cases:** This figure presents the results of the Gene Set Enrichment Analysis (GSEA) using Hallmark gene sets for genes that are common between two comparative cases: Case 1 and Case 2. It highlights significant biological pathways that are consistently implicated across different sample sets, elucidating potential mechanisms of lymph node metastasis in breast cancer.
